# High-quality assembly and methylome of a Tibetan wild tree peony genome (*Paeonia ludlowii)* reveal the evolution of giant genome architecture

**DOI:** 10.1093/hr/uhad241

**Published:** 2023-11-10

**Authors:** Pei-Xuan Xiao, Yuanrong Li, Jin Lu, Hao Zuo, Gesang Pingcuo, Hong Ying, Fan Zhao, Qiang Xu, Xiuli Zeng, Wen-Biao Jiao

**Affiliations:** National Key Laboratory for Germplasm Innovation & Utilization of Horticultural Crops, Huazhong Agricultural University, Wuhan 430070, China; Hubei Hongshan Laboratory, Wuhan 430070, China; Qinghai-Tibet Plateau Fruit Trees Scientific Observation Test Station (Ministry of Agriculture and Rural Affairs), Lhasa, Tibet 850032, China; Institute of Vegetables, Tibet Academy of Agricultural and Animal Husbandry Sciences, Lhasa, Tibet 850002, China; National Key Laboratory for Germplasm Innovation & Utilization of Horticultural Crops, Huazhong Agricultural University, Wuhan 430070, China; Hubei Hongshan Laboratory, Wuhan 430070, China; Qinghai-Tibet Plateau Fruit Trees Scientific Observation Test Station (Ministry of Agriculture and Rural Affairs), Lhasa, Tibet 850032, China; Qinghai-Tibet Plateau Fruit Trees Scientific Observation Test Station (Ministry of Agriculture and Rural Affairs), Lhasa, Tibet 850032, China; Institute of Vegetables, Tibet Academy of Agricultural and Animal Husbandry Sciences, Lhasa, Tibet 850002, China; Qinghai-Tibet Plateau Fruit Trees Scientific Observation Test Station (Ministry of Agriculture and Rural Affairs), Lhasa, Tibet 850032, China; Institute of Vegetables, Tibet Academy of Agricultural and Animal Husbandry Sciences, Lhasa, Tibet 850002, China; Qinghai-Tibet Plateau Fruit Trees Scientific Observation Test Station (Ministry of Agriculture and Rural Affairs), Lhasa, Tibet 850032, China; Institute of Vegetables, Tibet Academy of Agricultural and Animal Husbandry Sciences, Lhasa, Tibet 850002, China; National Key Laboratory for Germplasm Innovation & Utilization of Horticultural Crops, Huazhong Agricultural University, Wuhan 430070, China; Hubei Hongshan Laboratory, Wuhan 430070, China; Qinghai-Tibet Plateau Fruit Trees Scientific Observation Test Station (Ministry of Agriculture and Rural Affairs), Lhasa, Tibet 850032, China; Institute of Vegetables, Tibet Academy of Agricultural and Animal Husbandry Sciences, Lhasa, Tibet 850002, China; National Key Laboratory for Germplasm Innovation & Utilization of Horticultural Crops, Huazhong Agricultural University, Wuhan 430070, China; Hubei Hongshan Laboratory, Wuhan 430070, China

## Abstract

Tree peony belongs to one of the Saxifragales families, Paeoniaceae. It is one of the most famous ornamental plants, and is also a promising woody oil plant. Although two Paeoniaceae genomes have been released, their assembly qualities are still to be improved. Additionally, more genomes from wild peonies are needed to accelerate genomic-assisted breeding. Here we assemble a high-quality and chromosome-scale 10.3-Gb genome of a wild Tibetan tree peony, *Paeonia ludlowii*, which features substantial sequence divergence, including around 75% specific sequences and gene-level differentials compared with other peony genomes. Our phylogenetic analyses suggest that Saxifragales and Vitales are sister taxa and, together with rosids, they are the sister taxon to asterids. The *P. ludlowii* genome is characterized by frequent chromosome reductions, centromere rearrangements, broadly distributed heterochromatin, and recent continuous bursts of transposable element (TE) movement in peony, although it lacks recent whole-genome duplication. These recent TE bursts appeared during the uplift and glacial period of the Qinghai–Tibet Plateau, perhaps contributing to adaptation to rapid climate changes. Further integrated analyses with methylome data revealed that genome expansion in peony might be dynamically affected by complex interactions among TE proliferation, TE removal, and DNA methylation silencing. Such interactions also impact numerous recently duplicated genes, particularly those related to oil biosynthesis and flower traits. This genome resource will not only provide the genomic basis for tree peony breeding but also shed light on the study of the evolution of huge genome structures as well as their protein-coding genes.

## Introduction

Tree peony is one of the famous ornamental plants in Chinese culture with its beautiful flower and elegant fragrances. Wild peonies were domesticated and cultivated in China around 1500 years ago, then introduced into other East Asian countries in the Tang Dynasty, and later into Europe and North America during the 18 and 19th centuries. Now, more than 8000 cultivars are widely distributed in the world [1]. The production and markets of cut peony flowers have increased significantly in the past 30 years [2]. Apart from its ornamental value, tree peony is a candidate woody oil crop as its seeds are rich in unsaturated fatty acids like oleic acid (C18:1^Δ9^, OA), linoleic acid (C18:2^Δ9,12^, LA), and α-linolenic acid (C18:3^Δ9,12,15^, ALA) [3, 4]. Besides, the dry root of peony has been used in traditional Chinese medicine for cardiovascular, extravasated blood, and other diseases as it contains large amounts of paeoniflorin and paeonol compounds [5–7]. Tree peony, which was phylogenetically put into Ranunculaceae, is now classified in the family Paeoniaceae of the order Saxifragales. Paeoniaceae only has one genus, *Paeonia*, including around 35 wild species, which can be further grouped into three sections: *Moutan* (all woody peonies), *Onaepia* (all herbaceous peonies in the New World), and *Paeonia* (all herbaceous peonies in the Old World) [1, 8–10].

In recent years, rapid advancements of genomic technologies have greatly promoted research on the molecular breeding and functional genomics of peony [11–15]. To accelerate genomic-assisted breeding and cultivar improvement using wild genetic resources, a high-quality reference genome sequence of the wild tree peony is required. However, assembling the peony genome is greatly challenging as it is giant-sized (>10 Gb), with relatively huge chromosomes (2*n* = 2*x* = 10), and has a high fraction of repetitive sequences. Previous studies have released two tree peony genomes for the cultivar *Paeonia suffruticosa* [16] and the wild *P. ostii* [12]. As great divergences in phenotype and ecological habits exist in *Paeonia* [1], more peony genomes, especially from other sections, or subsections, of *Paeonia*, should provide more insights into its population history and benefit research on genomics-assisted breeding in peony.

Most wild *Paeonia* species mainly inhabit the temperate regions of the Northern Hemisphere, while some wild tree peonies, like *P. ludlowii*, have strict environmental requirements. *Paeonia ludlowii* is narrowly distributed in Linzhi (in Tibet) below an elevation of ~3000 m. Wild *P. ludlowii* was first discovered by Ludlow and Sherriff in 1936, and later was classified as a new species [1, 8, 9]. Only six wild *P. ludlowii* populations have been found so far [17]. One recent study has further characterized the genetic structure of the wild *P. ludlowii* population with RAD-sequencing [18]. Due to its narrow distribution and disturbances from human activities, *P. ludlowii* has become an endangered species. Unlike other wild *Paeonia* species, *P. ludlowii* has the rare large pure yellow flowers (Supplementary Data Fig. S1), making it a valuable genetic resource for breeding. However, very few cases of hybrid breeding using *P. ludlowii* have been mentioned until now [1]. Apart from the ornamental value, recent studies have also revealed the content of abundant unsaturated fatty acids in seeds and potential medical compounds in the roots and seeds of *P. ludlowii* and other peony species [3, 5, 19]. In addition, some studies on *P. ludlowii* have focused on seed biology, like endosperm abortion or seed abortion, as the natural reproduction of *P. ludlowii* is limited due to its low fecundity [20].

However, genomic or transcriptomic-level characterization in these studies has been constrained by the lack of a reference *P. ludlowii* genome. Here we report a chromosome-scale 10-Gb genome of *P. ludlowii* with high-quality transcriptome and methylomes. Our assembly presents much better assembly contiguity and completeness. We identify great sequence divergences at different levels between *P. ludlowii* and other *Paeonia* genomes. We reconstruct the phylogeny of peony and 19 other angiosperm genomes to provide new support for the phylogenetic relationship across rosids, Saxifragales, Vitales, and asterids. Further comparative genomic analyses reveal the role of chromosome rearrangements and the centromere during the evolution of *P. ludlowii* giga-chromosomes. Besides, we demonstrate the impact of bursts of transposable element (TE) transposition and DNA methylation on genome size expansion and gene duplication, as well as genes related to traits of flower color, scent, and seed oil.

## Results

### Chromosome-level assembly and annotation of *P. ludlowii*

**Figure 1:**
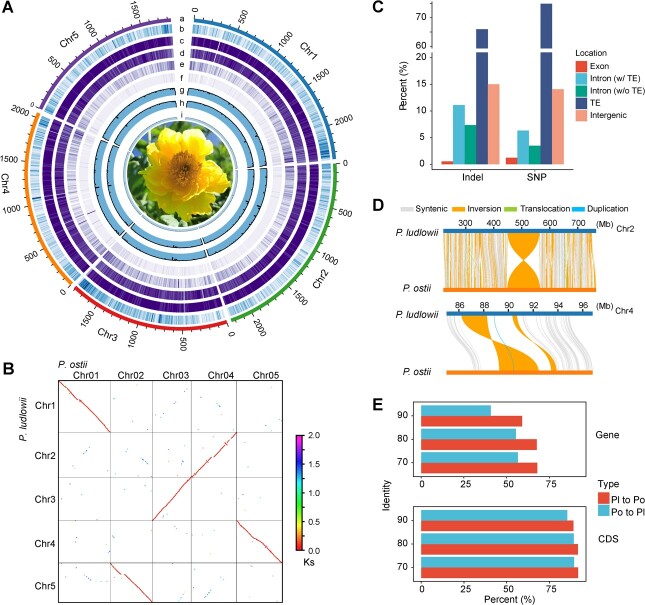
Genomic characteristics of *P. ludlowii* and sequence variations between tree peony genomes. **A** Tracks of the Circos plot indicate the overall characteristics of the *P. ludlowii* genome with a sliding window of 10 Mb. (Track a) Chromosome ideogram; tracks b–f, density of genes, TEs, Gypsy, Copia, and DNA transposons. (Tracks g–i) Density of DNA methylation levels in CG, CHG, and CHH contexts. **B** Dot plot of sequence alignments between *P. ludlowii* and *P. ostii*. **C** SNPs and indels in different genomic regions between *P. ludlowii* and *P. ostii*. **D** Example of inversion between *P. ludlowii* and *P. ostii*. **E** Percentage of *P. ostii* or *P. ludlowii* genes that could be aligned to the *P. ludlowii* or *P. ostii* genome with different cutoff values of sequence identity. Pl, *P. ludlowii*; Po, *P. ostii*.

To obtain the genome sequences of *P. ludlowii*, we generated 320.1 Gb (~30×) PacBio long high-fidelity (HiFi) reads, 833.8 Gb (~78×) Illumina short paired-end reads and 1001.7 Gb (~94×) Hi-C reads (Supplementary Data Table S1). The genome sequences were assembled by combining the *de novo* assembled contigs resulting from two assemblers, hifiasm [21] and HiCanu [22]. The assembled contigs featured an N50 (L50) of 1.15 Mb (2790), which is ~23- or 4-fold of the contig N50 value of *Paeonia suffruticosa* [16] or *Paeonia ostii* [12]. The estimated genome size of *P. ludlowii* is ~10.6 Gb (Supplementary Data Fig. S2, Supplementary Data Table S2), smaller than those of *P. suffruticosa* (13.66 Gb) and *P. ostii* (12.76 Gb). Our assembly has a total length of 11.3 Gb (Supplementary Data Table S3). The longer assembled sequences might be due to the redundancy of highly similar centromeric or repetitive regions. A total of 10.33 Gb (91.68%) contigs were successfully anchored into five pseudochromosomes using Hi-C-based scaffolding methods (Fig. 1A, Supplementary Data Table S4, Supplementary Data Fig S3). The final assembly showed a Benchmarking Universal Single-Copy Orthologs (BUSCO) completeness value of 98.5%, higher than both *P. suffruticosa* (61.2%) and *P. ostii* (94.4%) (Supplementary Data Table S5). The *k*-mer-based completeness of *P. ludlowii* is 97.0%, close to the BUSCO value. Besides, 99.2% of the Illumina short reads and 99.97% of the HiFi reads could be mapped to the assembly. These results together suggest that our *P. ludlowii* assembly presents great improvements in quality.

To improve the gene annotation of the *P. ludlowii* genome, we produced 154.4-Gb Illumina RNA-seq reads from six different tissues, including roots, fruits, petals, buds, leaves, and branches (Supplementary Data Table S6). By integrating the evidence of RNA-seq transcripts, alignments of protein sequences, and *ab initio* gene prediction, we annotated 46 582 high-confidence protein-coding gene models, with 99.35% of them supported by RNA-seq reads, orthologous proteins, or functional annotation (Supplementary Data Tables S7 andS8). The annotation of fewer protein-coding genes in our peony genome compared with others is mainly due to the different control of high-confidence predictions and interspecies genome divergence. Moreover, we predicted 76 000 non-coding genes, including 53 959 microRNAs, 13 386 rRNAs, 3771 tRNAs, 2725 snRNAs, 1429 snoRNAs, and 730 lncRNAs (Supplementary Data Table S9).

### Substantial divergence between *P. ludlowii* and *P. ostii* genomes

To investigate the sequence divergence between *P. ludlowii* and *P. ostii* genomes, we performed synteny analysis, whole-genome sequence comparison, and gene-level alignments. Overall, the two genomes show large-scale synteny except for a few inversions (Fig. 1B). Aligning two 10-Gb-level giant genomes is substantially challenging due to the huge chromosome size, high repeat content, and memory cost. We partitioned each chromosome into reasonable sub-chromosomes according to the gene-level synteny boundary, then aligned the syntenic sub-chromosomes with the tool MUMmer [23]. Surprisingly, only around 10.0% (1.03 Gb) of the whole genome could be aligned with sequence identity >90%. Relaxing the cutoff for the minimal value of sequence identity to 80% led to 16.3% more aligned regions, while further lowering the cutoff captured more alignments marginally (Supplementary Data Table S10). Approximately 88.7% of unaligned regions were occupied by TEs, especially long terminal repeat (LTR) retrotransposons, suggesting that lineage-specific accumulation or faster divergence of TE exists in *Paeonia*. In total, we identified 34 123 611 SNPs and 4 965 543 indels (<50 bp) in 2.71 Gb aligned regions with alignment identity >80% (Fig. 1C). We also found 373 547 large structural variations (SVs), including 11 223 deletions, 10 975 insertions, 4894 inversions, and 346 455 translocations. For example, 360 inversions over 1 Mb were detected (example shown in Fig. 1D). Another tree peony genome, *P. suffruticosa*, without chromosome-level assembly, has a relatively higher alignment rate with *P. ostii* than with *P. ludlowii* (Supplementary Data Table S11).

Apart from whole-genome sequence alignment, we also performed gene-level alignment. We found that 32.18% of *P. ludlowii* genes could not be aligned to the *P. ostii* genome or showed lower sequence similarity (identity <70%), while a higher fraction of *P. ostii* genes were not aligned (Fig. 1E, Supplementary Data Table S12). However, the coding sequence-level alignments demonstrated that ~90% of *P. ludlowii* genes could be aligned to *P. ostii* and vice versa, implying that these sister peony genomes have a much higher differentiation in intronic regions. Further comparisons of their ortholog gene pairs revealed that such differentiations are mainly (87.6%) caused by species-specific TE insertions in introns (Supplementary Data Fig. S4). On the whole, these comparisons together suggested that great sequence divergence occurred between these sister peony genomes, which might challenge the intra-section hybrid breeding of *Paeonia*.

### Phylogenomic analyses of Saxifragales, Vitales, rosids, and asterids

**Figure 2:**
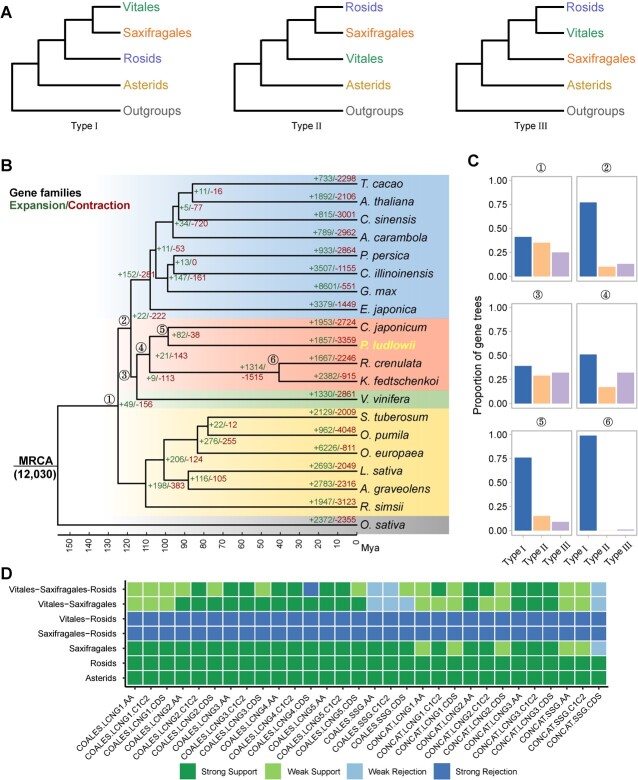
Phylogenomic analyses of Saxifragales, Vitales, rosids, and asterids. **A** Three different topologies of phylogenetic relationships across Vitales, Saxifragales, rosids, and asterids. **B** Phylogenetic tree of 20 representative plant genomes inferred using IQ-TREE 2 [24] based on the alignment of coding sequences from 1215 orthologous LCN genes. Circled numbers 1–6 indicate which internal branches were examined for gene tree incongruence, as shown in **C**. Numbers on the branches indicate gene family expansion (left) and contraction (right). MRCA, most recent common ancestor; Mya, million years ago. **C** Gene tree incongruence with quartet support for the main topology (Type I), the first alternative topology (Type II), and the second alternative topology (Type III), which correspond to the three different topologies in **A**. **D** Visualization of phylogenetic inconsistency with the tool DiscoVista [25]. Each row indicates one hypothetical group tested with different datasets or methods. COALES, coalescent; CONCAT, concatenate; LCNG1/LCNG2/LCNG3/LCNG4/LCNG5, low-copy nuclear genes with at most one/two/three/four/five species orthologs have multiple copies; SSCG, single-copy genes; AA, amino acid; C1C2, codon 1st + 2nd positions; Strong Support, the clade is reconstructed with a support value >95%; Weak Support, the clade is reconstructed with a support value <95%; Weak Rejection, the clade is not recovered, but the alternative topology is not in conflict if poorly supported branches (<85%) are collapsed; Strong Rejection, the clade is not recovered, and the alternative topology is in conflict even when poorly supported branches (<85%) are collapsed.

The family Paeoniaceae belongs to the order Saxifragales. However, the phylogenetic relationship among Saxifragales, Vitales, rosid, and asterid clades remains controversial. According to previous studies [26], Saxifragales, Vitales, and rosids can be clustered into three different topologies (Fig. 2A). To resolve the phylogenetic relationship, we initially collected 1215 orthologous low-copy nuclear (LCN) genes from *P. ludlowii*, 18 other core eudicot genomes (8 rosids, 6 asterids, 3 Saxifragales, 1 Vitales), and 1 outgroup from the monocot *Oryza sativa* (Supplementary Data Table S13; see Materials and methods). A highly supported species tree was obtained based on the maximum-likelihood method with concatenated alignments of protein-coding regions (Fig. 2B and C). This phylogenetic tree showed that Saxifragales was sister to Vitales, rather than the rosids. Saxifragales + Vitales was sister to rosids, and together with rosids, the sister to asterids. The same phylogenetic topology was revealed by the coalescent-based phylogenetic analysis of each gene tree (Supplementary Data Fig. S5).

In addition, we applied both the concatenated and coalescent-based methods to reconstruct the phylogenetic tree based on the alignments of protein sequences and the first and second codons. Such different alignment methods yielded highly similar phylogenetic topologies as well (Fig. 2D, Supplementary Data Fig. S5). Besides, we selected another four different orthologous LCN gene group sets, including 301, 710, 1768, and 2333 groups, based on different criteria (see Materials and methods for details). Phylogenetic analyses of these different gene sets almost all revealed similar tree topologies (Supplementary Data Figs S6–S9). Furthermore, to account for the impact of different outgroups, we identified 1305 and 1124 orthologous LCN genes with alternative outgroups of *Aquilegia coerulea* and *Buxus sinica*, respectively (Supplementary Data Table S14). Again, nearly all phylogenetic trees had the same topology as the former tree (Fig. 2D, Supplementary Data Fig. S10). Finally, we inferred the divergence time of core eudicot lineages based on the string set of 1215 LCN genes and age calibrations from fossils (Supplementary Data Fig. S11 and Materials and methods). The split between Saxifragales and Vitales was predicted to occur around 114.84 million years ago (Mya), which was consistent with some previous reports [27, 28].

### Chromosome karyotype evolution without recent whole-genome duplication

**Figure 3:**
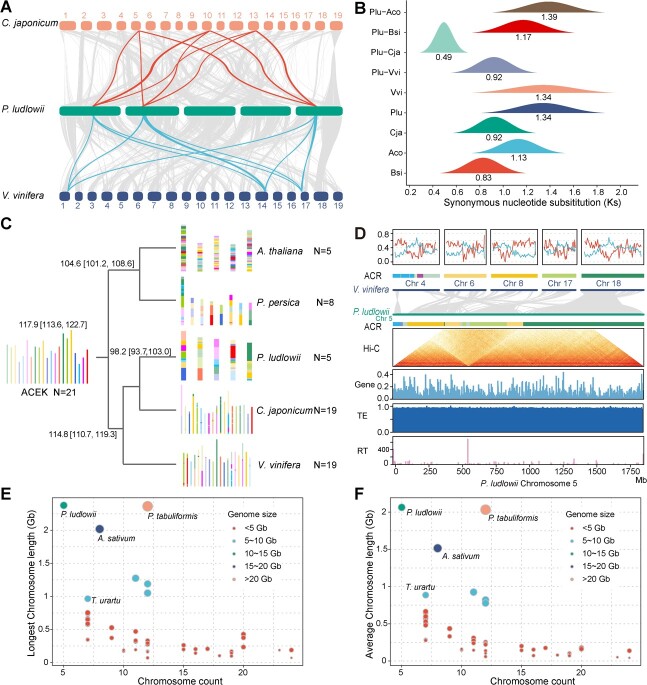
Genome and chromosome karyotype evolution of *P. ludlowii*. **A** Syntenic blocks between *P. ludlowii*, *C. japonicum*, and *V. vinifera*. An example of a 3:3 syntenic block relationship is highlighted with red and blue links. **B***K*_s_ distribution of orthologous and paralogous genes. Orthologs are identified between *P. ludlowii* (Plu) and *A. coerulea* (Aco), *B. sinica* (Bsi), *C. japonicum* (Cja), and *V. vinifera* (Vvi). Paralogs are identified in *V. vinifera*, *P. ludlowii*, *C. japonicum*, *A. coerulea*, and *B. sinica*. The numbers under the density curves represent the mean values. **C** Chromosome karyotype evolution of *V. vinifera*, *P. ludlowii*, *C. japonicum*, *P. persica*, and *A. thaliana*. Colors represent the different segments evolving from the common ancestral chromosome karyotype (ACEK = 21). ACEK, ancestral core eudicot karyotype. **D** Chromosome karyotype comparisons between *P. ludlowii* and grape. From top to bottom, the figure displays gene (red line) and TE (blue line) density (%) of a 500-kb sliding window along chromosomes as well as composition of ancestral chromosome karyotype (ACR) of the grape genome, syntenic relationship with chromosome 5 of *P. ludlowii*; and composition of ancestral chromosome karyotype (ACR), Hi-C contact map, gene density, TE density, and TR (tandem repeat) number of *P. ludlowii* chromosome 5. **E**, **F** Longest (E) and average (F) chromosome lengths of *P. ludlowii* and 45 other representative plants with genome sizes >1 Gb. The size and color of the dots indicate the size of the genome.

We next investigated the impact of whole genome duplication (WGD) on the peony genome based on the analyses of syntenic blocks and synonymous nucleotide substitutions (*K*_s_) with homologous genes. We detected 320 intra-genome syntenic blocks in the comparisons of the *P. ludlowii* genome itself, which mainly indicated a 1:3 ratio (Supplementary Data Fig. S12). Further syntenic comparisons with the phylogenetically close species grape and *Cercidiphyllum japonicum*, which neither have experienced a recent WGD event, both showed an enrichment in the 3:3 relationship (Fig. 3A, Supplementary Data Fig. S12). In addition, the synteny analysis revealed an enrichment of a 3:2 ratio between *P. ludlowii* and *B. sinica*, which has experienced a WGD event after divergence with other core eudicots (Supplementary Data Fig. S12). These results together implied that no recent or lineage-specific WGD event occurred in Paeoniaceae after divergence with Cercidiphyllaceae. Besides, the distribution of *K*_s_ values of gene pairs in *P. ludlowii*–*P. ludlowii* syntenic blocks only showed one peak around 1.34 (Fig. 3B), which corresponded to the ancient gamma WGD event shared by core eudicots [29].

Unlike other published Saxifragales genomes, like *C. japonicum* and *Kalanchoë fedtschenkoi*, which have 19 and 17 chromosome pairs, the peony genome only retains five pairs of chromosomes, implying that large chromosome rearrangements occurred after divergence from *C. japonicum*. We reconstructed the ancestral chromosome karyotypes of Saxifragales and superrosids based on syntenic analysis across the genomes of *P. ludlowii* and *C. japonicum* as representatives of Saxifragales, *Prunus persica*, and *Arabidopsis thaliana* as representatives of rosids, and grape as the representative of the sister order of Saxifragales. The evolutionary trajectories of the chromosome karyotype revealed that many more chromosomal fissions and chromosome fusions might have occurred in *P. ludlowii* compared with its sister species, *C. japonicum* (Fig. 3C).

The greatly reduced chromosome number also suggests that many more centromere changes, like centromere loss and/or repositioning, might happen in the *P. ludlowii* genome. Syntenic analyses between *P. ludlowii*, grape, and other Saxifragales genomes indicated ancestral centromere loss and repositioning (Fig. 3D, Supplementary Data Fig. S13). Additionally, we predicted the potential position of centromeres based on tandem repeat annotation [30]. We found one 158-bp tandem repeat unit that might be the potential centromeric-specific repeat sequence (Supplementary Data Fig. S13, Supplementary Data Table S15). Such repeats tandemly form higher-order repeat structures in all chromosomes. However, on chromosome 4 only, they span a large region at 929–965 Mb, consistent with the Hi-C contact maps (Supplementary Data Fig. S13). This implies that the peony genome might still be in the process of accumulating centromeric repeats to form new centromeres after centromere loss. Moreover, the almost even distribution of transposons along chromosomes indicated that the peony genome had no obvious boundary for the pericentromeric regions. However, the grape genome and other Saxifragales genomes show a clear peak of TE density around centromeres (Fig. 3D). These results together might suggest the peony genome presents broadly distributed pericentromeric regions and has experienced much centromere loss and neocentromere formation, along with chromosome number reduction, during genome evolution.

### Genome expansion due to continuous bursts of active transposable element movements

Apart from the frequent chromosome reductions that arose in *P. ludlowii* relative to the ancestral genome, the chromosome size of peony has greatly expanded. *Paeonia ludlowii* harbors the largest average chromosome size compared with other giant genomes sequenced so far (>10 Gb) (Fig. 3E and F). The genome of *P. ludlowii* consists of 10.34 Gb (92.0%) repetitive sequences, including 9.83 Gb (87.4%) of TEs (Supplementary Data Table S16). Most (~61.6%) of the genome comprises LTR retrotransposons. For LTR retrotransposons, *Ty1/Copia* elements only account for 5.6%, while *Ty3/Gypsy* elements occupy 38.0% of the whole genome (Supplementary Data Table S16). Additionally, 18.1% of the genome is occupied by non-autonomous LTR retrotransposons.

**Figure 4:**
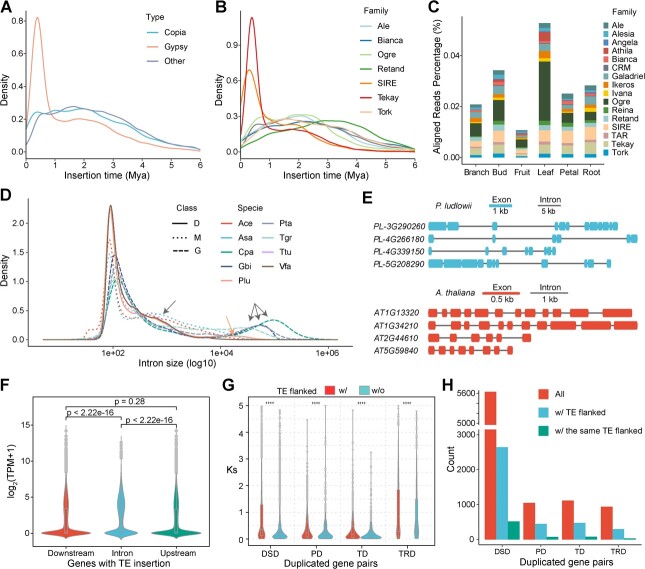
Expansion of genome size and recently duplicated gene pairs driven by bursts of active TE transposition. **A** Estimation of insertion time for intact LTRs of Copia, Gypsy, and other LTRs. **B** Estimation of insertion time for intact LTR in the largest subfamilies. **C** Estimation of TE activity based on the mapped RNA-seq reads across six tissues. **D** Intron size distribution of 12 representative plant genomes with huge genome sizes. The relatively lower peaks, shown by arrows, indicate intron size expansion. D, eudicots; M, monocots; G, gymnosperms; Ace, *Allium cepa*; Asa, *Allium sativum*; Cpa, *Cycas panzhihuaensis*; Gbi, *Ginkgo biloba*; Plu, *Paeonia ludlowii*; Pta, *Pinus tabuliformis*; Tgr, *Torreya grandis*; Ttu, *Triticum turgidum*; Vfa, *Vicia faba*. **E** Gene structure of the top four longest genes of *P. ludlowii* and their orthologs in *A. thaliana*. **F** Expression comparison across genes with TEs at their introns, upstream and downstream. **G***K*_s_ distribution of duplicated gene pairs with or without TEs in flanking sides across groups of TD (tandem duplication), PD (proximal duplication), DSD (dispersed duplication), and TRD (transposed duplication). **H** Number of all, TE-flanked, and same-TE-flanked recently duplicated gene pairs for each duplication group.

Based on the analysis of the sequence divergence of intact LTRs, we predicted two burst events of *Ty3/Gypsy* transposition (Fig. 4A). The more recent burst event occurred around 0.4 Mya, which was probably caused by the rapid transposition of the Tekay family (Fig. 4B, Supplementary Data Table S17). However, such a recent burst event was not reported in the *P. ostii* genome, perhaps due to the failure of assembling highly similar LTRs or just a specific burst event in *P. ludlowii* [12]. This Tekay family presents ~350 000 copies with a total length of 1.39 Gb in *P. ludlowii*. Besides, the earlier burst event of Gypsy elements (such as Retand and Ogre) appeared at 1–4 Mya (Fig. 4B, Supplementary Data Table S17). Additionally, several TE families contributed to the continuous transposition of Copia and non-autonomous LTRs around 1.5–2.5 Mya (Fig. 4B). The earlier burst of TE activity appeared between the late Miocene and Pliocene, when dramatic uplift and climate changes occurred in the Qinghai–Tibet Plateau (QTP) [31], consistent with another plant species, *Crucihimalaya himalaica*, also inhabiting the QTP [32]. The more recent TE burst was close to a glacial period (0.6–0.7 Mya) [33]; these results together suggest that the TE proliferation might have contributed to the speciation and adaptation of *Paeonia* in the QTP. We further analyzed the solo LTRs, which are frequently derived from unequal recombination of intact LTRs. The ratio of solo:intact LTRs in *P. ludlowii* was much higher than in other eudicots (Supplementary Data Fig. S14), implying that a relatively dramatic elimination of LTRs might exist in peony to counteract active TE proliferation.

### Impact of transposable element movements on protein-coding genes

Such continuous expansion of TEs may suggest that some TE families still move actively within the genome. To examine their expression level, we checked the RNA-seq reads mapped to different TE families. As expected, the number of TE-mapped reads was positively correlated to the content of TE families (Fig. 4C). Active TE movements can also greatly affect the structure and expression of genes when they occur in or around genic regions. A total of 552 862 TE elements were located in introns of 40.9% (19 035) of 46 582 intron-containing protein-coding genes (Supplementary Data Table S18). Compared with LTRs, DNA transposons tended to insert into more genes, although they have a relatively lower number in the whole genome (Supplementary Data Table S18). TE insertions resulted in an expansion of the average size of genes (~6.24 kb) and introns (~1.35 kb) compared with other Saxifragales and Vitales genomes (Fig. 4D, Supplementary Data Table S19). For example, 349 genes with TE insertion were longer than 100 kb (examples shown in Fig. 4E). Around one-third (107) of them could be expressed and had similar exon numbers to their orthologs in *A. thaliana*. Enlarged comparisons of intron size across more genomes from different plant clades indicated a relatively lower peak at around 17.3 kb in peony and 0.4–0.6 kb in other giant angiosperm genomes, while other lower peaks occurred at 24–94 kb in gymnosperms (Fig. 4D). Besides, 396 986 TEs were within 2 kb upstream or downstream of 45 904 (98.5%) protein-coding genes (Supplementary Data Table S20). Moreover, expression of TE-inserted genes was significantly higher than that of genes without inserted or flanked TEs (Fig. 4F), suggesting that the active movements of TEs reshaped both the structure and expression of genes in peony.

As the *P. ludlowii* genome has a high proportion of TE content and no experience of recent WGD events, we also investigated whether TE movements boosted a recent burst of gene duplication in the *P. ludlowii* genome. We identified 53 983 duplicated gene pairs that were not due to WGD. Around 35.9% of them were flanked by TEs and tended to diverge faster than those without flanked TEs (Fig. 4G). The peak *K*_s_ values are around 0.3, implying a recent burst of duplicated genes (Supplementary Data Fig. S15), which was also indicated in TE-enriched Triticeae genomes. These recently duplicated gene pairs (*K*_s_ < 0.3) were classified into tandem (TD, 1114), proximal (PD, 1049), dispersed (DSD, 5630), and transposed (TRD, 938) categories (Fig. 4H, Supplementary Data Table S21). Approximately 32.2–47.0% of them were flanked by TEs, which are mainly from LTRs (19.2–28.3%). In addition, 3.62–9.25% of them were flanked by the same LTR families. As expected, a large fraction (28.6–35.5%) of these gene pairs have lost introns in at least one of them. Taken together, these results indicate that active TE movements, especially of retrotransposons, have played an important role in the evolution of recent gene duplication.

### Landscape of DNA methylation

**Figure 5:**
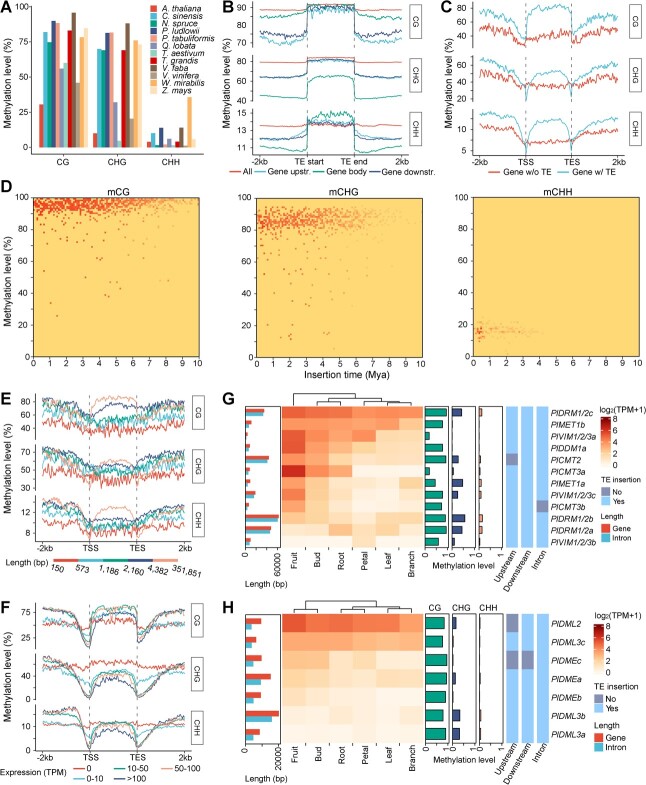
DNA methylation landscape of the *P. ludlowii* genome. **A** Genome-wide DNA methylation level of *P. ludlowii* and 12 other plant genomes (Supplementary Data Table S22) with high TE content (>60%) or genome size (>5 Gb). **B** CG, CHG, and CHH methylation patterns along all TEs, TEs located 2 kb upstream and downstream of genes, and TEs located in gene bodies. **C** Patterns of CG, CHG, and CHH DNA methylation in genes with and without TE-inserted introns. **D** DNA methylation level of CG, CHG, and CHH contexts in transposons with different insertion times. **E**, **F** Patterns of CG, CHG, and CHH DNA methylation in genes with different lengths (**E**) and expression levels (**F**). **G**, **H** Expression, DNA methylation, TE insertion, and sequence length of DNA methylation-related (**G**) and demethylation-related (**H**) genes. Expression level was quantified based on TPM (transcripts per million) and visualized as a heat map with hierarchal clustering.

TE activity is correlated with the level of DNA methylation. We next conducted whole-genome bisulfite sequencing to investigate the DNA methylation landscape of *P. ludlowii* (Fig. 5). The averages of whole-genome methylation levels across CG, CHG, and CHH nucleotide contexts were 89.94, 81.38, and 13.84%, respectively, which were higher than those for most currently available large (>5 Gb) or TE-rich (>60%) genomes (Fig. 5A, Supplementary Data Table S22). TEs were heavily methylated, with an average of 91.87% (CG), 84.03% (CHG), and 13.64% (CHH) at genome-wide level, while TEs in the upstream, downstream, and introns of protein-coding genes presented a relatively lower level of methylation (Fig. 5B). As in Chinese pine and faba bean genomes [34, 35], we have not found evidence supporting the idea that the TE methylation level was negatively correlated with the TE insertion time, as reported in some plant genomes (Fig. 5D).

The methylation level of protein-coding genes decreased considerably near the transcription start and end sites. Genes with TE-inserted introns displayed a greatly increased methylation level in all CG, CHG, and CHH contexts in gene bodies (Fig. 5C). Such an increase in gene body methylation on TE-inserted genes might explain their relatively higher expression (Fig. 4F, Supplementary Data Fig. S16). Likewise, larger genes tended to have higher methylation levels (Fig. 5E). Similar to the pattern in other plant genomes [34, 36], the average methylation level near the transcription starting sites was negatively correlated with the gene expression level (Fig. 5F). Compared with other Saxifragales and Vitales genomes, *P. ludlowii* has more gene copies of *DRM1/DRM2* involved in *de novo* methylation in all DNA contexts and of *DME* and *DML3* involved in DNA demethylation (Fig. 5G and H, Supplementary Data Fig. S17). We found at least one TE insertion in genic regions of nearly all genes involved in pathways of DNA methylation establishment and maintenance (Fig. 5G). Additionally, TE insertions were also found in all DNA demethylation pathway genes. Some of these TE-inserted genes showed high expression in different tissues or in some specific tissues. Taking these results together, TE-introduced copy and expression changes of methylation/demethylation-related genes might provide a basis for the high level of DNA methylation.

### Evolutionary impact on genes related to oil biosynthesis

**Figure 6:**
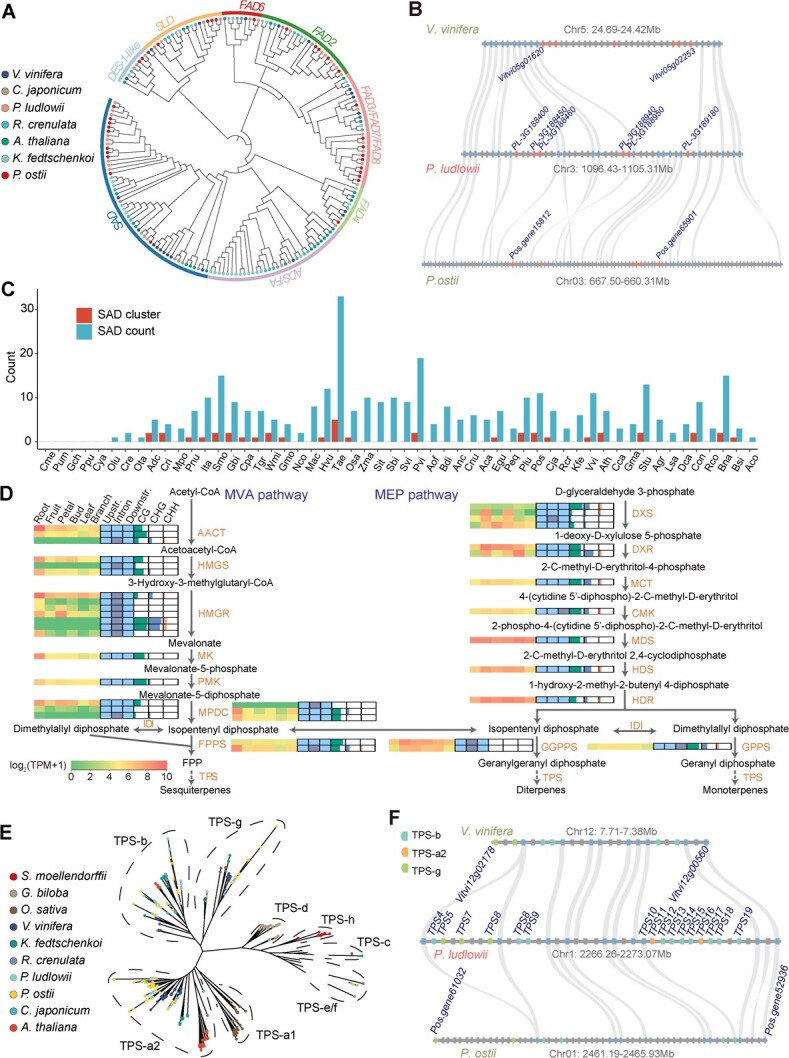
Impact of TE and DNA methylation on genes related to the biosynthesis of fatty acids and flower scent. **A** Phylogenetic analysis of the *FAD* gene family across *P. ludlowii* and six other plant genomes. Text in the outer circle and colors of the branches indicate the different subfamilies of FAD. **B** Micro-synteny in a region containing multiple *SAD* (red) genes between *P. ludlowii* and *P. ostii*, plotted by jcvi [37]. Orthologous genes (*SAD* excluded) are indicated with blue triangles and gray links. **C***SAD* gene cluster and count across 54 representative plant genomes (details in Supplementary Data Table S24). **D** Expression, DNA methylation, and duplication of genes potentially involved in the biosynthesis of terpenoids. Expression level is quantified based on TPM (transcripts per million) and visualized as heat maps. The light blue and gray rectangles indicate genes with (blue) or without (gray) TE insertion at upstream, downstream, and intron positions. DNA methylation levels in CG, CHG, and CHH sequence contexts are shown in horizontal histograms. **E** Phylogenetic analysis of the TPS gene family across *P. ludlowii* and nine other plant genomes. **F** Locally syntenic alignment of one tandem TPS gene cluster among *P. ludlowii*, *P. ostii*, and grape (*V. vinifera*) genomes. Different groups of TPS genes are indicated by colored rectangles. Orthologous genes (TPS excluded) are indicated with blue rectangles and gray links. Other non-TPS genes without orthologs are shown by gray rectangles.

Seeds of tree peony have been characterized by abundant unsaturated fatty acid and a high content of ALA. Our GC–MS experiments showed that OA in *P. ludlowii* was much higher (30.3–48.0%) than LA (12.1–19.5%) and ALA (25.5–37.9%), while the ALA content was highest in *P. ostii* (Supplementary Data Table S23). Gene family clustering and phylogenetic analysis with seven representative plant genomes revealed copy expansion of *SAD* (*stearoyl-ACP desaturase*, 10 genes), *FAD2* (*fatty acid desaturase 2*, 7 genes), and *FAD3* (*fatty acid desaturase 3*, 2 genes) (Supplementary Data Fig. S18, Fig. 6A), and copy divergence of these genes between *P. ludlowii* and *P. ostii* (Supplementary Data Fig. S19). Some fatty acid biosynthesis genes are tandemly clustered in *P. ludlowii*, such as the *SAD* genes (Supplementary Data Fig. S20). Local synteny analysis showed structural divergence at one tandem *SAD* gene cluster at chromosome 3, which was associated with C18:0/C18:1^△9^, as reported previously [12] (Fig. 6B). Such copy expansion and divergence might provide the genomic basis for the differences in OA and ALA content between *P. ludlowii* and *P. ostii*. However, more functional experiments in the future should demonstrate the mechanism underlying this evolutionary divergence in unsaturated fatty acid content. Such a *SAD* gene cluster was found in some angiosperms, Gymnospermae, Lycopodiophyta, Bryophyta, and Pteridophyta, but not in Chlorophyta and Rhodophytina (Fig. 6C, Supplementary Data Table S24). Gene syntenic analysis indicated no obvious collinear arrangement of the *SAD* cluster and their flanking genes (Fig. 6B), indicating an independent origin potentially driven by TE-introduced tandem duplication in different clades of Embryophyta.

### Evolutionary impacts on genes related to flower color and scent

As *P. ludlowii* has rare pure yellow flowers, we further characterized the candidate genes in the biosynthesis pathway of flavonoids, which constitute the flower pigment in tree peony [15]. We found that all these candidate genes in *P. ludlowii* contained TE insertion and, compared with *P. ostii*, copy number differences were found in genes for phenylalanine ammonia lyase (PAL), chalcone synthase (CHS), chalcone isomerase (CHI), anthocyanidin synthase (ANS), and flavonol synthase (FLS) (Supplementary Data Fig. S21). Our transcriptomic data indicated that the expression of genes ANS, dihydroflavonol 4-reductase (DFR), and UDP-glucose flavonoid 3-*O*-glucosyltransferase (UFGT), which are involved in the conversion of leucocyanidin to anthocyanins, did not show higher expression in petals. This is similar to the previous finding in ‘High Noon’, a peony cultivar with yellow flowers. However, two copies of the gene FLS, responsible for flavonol biosynthesis, were expressed at high levels in *P. ludlowii*. Interestingly, the other two copies with lower expression in petals showed a higher CG methylation level, perhaps due to TE insertions. In addition, one *P. ludlowii* homologous MYB transcription factor (*PL-3G145120*) of *PsMYB4*, which potentially interacts with bHLH transcription factors to reduce the synthesis of anthocyanins [15], showed high expression in buds and petals. For another MYB transcription factor, *PsMYB111* [15], which correlates with the increase in flavonols in flowers, one (*PL-3G267120*) of its homologs in *P. ludlowii* also showed higher expression in petals.

Gene family clustering indicated that genes related to terpene biosynthesis were frequently duplicated in the *P. ludlowii* genome. In plants, terpenoids are synthesized via the mevalonate (MVA) pathway and the 2-C-methyl-d-erythritol 4-phosphate (MEP) pathway. Genes (such as *AACT*, *HMGS*, *HMGR*, *DXS*, and *DXR*) involved in these pathways are duplicated in different ways (Fig. 6D). TEs are found in the intron, 2 kb upstream or 2 kb downstream of nearly all these duplicated and non-duplicated genes. Besides, we annotated 75 and 65 terpene synthase (TPS) genes in *P. ludlowii* and *P. ostii*, respectively (Supplementary Data Fig. S22). Compared with eight other representative plant genomes, phylogenetic analysis showed increased gene copies of TPSs in the subfamilies TPS-a2, TPS-b, and TPS-g for tree peony (Fig. 6D). Most of these increased copies locate in tandem duplication regions with higher TE percentages (Supplementary Data Fig. S23), indicating that the active TE movements might contribute to the origin of tandem TPS clusters. Although similar numbers of TPS clusters were present in *P. ludlowii* and *P. ostii* genomes, comparable divergence was found in the structure of the syntenic TPS clusters (Supplementary Data Fig. S24). For example, *P. ludlowii* has 16 copies in one TPS cluster in chromosome 1 (2266.26–2273.07 Mb), while both *P. ostii* and grape genomes only have 6 copies in such a region (Fig. 6F).

In *P. ludlowii*, 50 (66.7%) TPS genes have TE-inserted introns, and all of them have TE insertion in their 2 kb upstream or downstream regions (Supplementary Data Fig. S23). All those TPS genes with relatively higher expression have TE-inserted introns and higher levels of gene body methylation. For example, genes like *PlTPS45*, *PlTPS46*, *PlTPS47*, *PlTPS11*, and *PlTPS13* showed much higher expression levels in petal. Their homologous genes have been previously reported as candidate TPS genes for the specific monoterpene linalool in subsection Delavayanae (including *P. ludlowii*) [14], implying that TE and perhaps TE-introduced DNA methylation might play a role in the genetic basis of flavor evolution in tree peony.

## Discussion

Although there are more than 8000 peony cultivars, limited wild resources are used in breeding [1]. In this study we report a chromosome-level assembly of one wild tree peony, *P. ludlowii*. With PacBio HiFi and Hi-C sequencing technologies, we obtained a genome assembly with higher quality in terms of assembly contiguity and completeness compared with other published *Paeonia* genomes [12, 16]. Besides, we found large sequence and structural variations between our *P. ludlowii* genome and other *Paeonia* genomes. This high divergence between sister species suggests that more genome assemblies from other *Paeonia* species will provide deeper insights into the speciation and domestication of peonies and also contribute to the hybrid breeding of peonies.

As the sequencing cost has reduced in recent years, many huge (e.g. >5 Gb) plant genomes have been sequenced. These genomes phylogenetically belong to different clades, including eudicots [e.g. faba bean (11.90 Gb) [35]], monocots [e.g. bread wheat (15.4–15.8 Gb) [38], *Allium sativum* (16.2 Gb) [39]], gymnosperms [e.g. Chinese pine (25.4 Gb) [34], *Torreya grandis* (19.1 Gb) [40], *Cycas panzhihuaensis* (10.5 Gb) [41], *Taxus chinensis* (10.2 Gb) [42]], and ferns [e.g. *Ceratopteris richardii* (7.5 Gb) [43], *Alsophila spinulosa* (6.2 Gb) [44]]. Their genome size boosts are mainly triggered by events of WGD or polyploidization, and bursts of TE proliferation. Similar to those size-expanded genomes without recent lineage-specific WGD, *P. ludlowii* has experienced recurrent bursts of transposon movement, especially retrotransposon accumulation. However, unlike some huge plant genomes such as the faba bean, a higher solo:intact LTR ratio was found in *P. ludlowii*, indicating that a relatively faster removal mechanism may counter the TE accumulation. Besides, the high DNA methylation level in TEs also suggests that TE accumulation is under strong suppression or that the DNA methylation might not be effective enough to suppress TE proliferation. Similarly high levels of DNA methylation were also found in giant genomes such as faba bean, Chinese pine, and *Torreya grandis* [35, 40]. Thus, genome size expansion might be dynamically affected by a complex interaction among TE proliferation, TE removal, and DNA methylation silencing. Besides, the chromosome rearrangement and centromere loss and reposition also shape the giga-size chromosomes of *P. ludlowii*.

Apart from the effects on genome and chromosome structures, the active TE movements together with DNA methylation also impact the exon–intron structure and expression of a large number of genes. Moreover, TE reposition apparently correlates with the burst of recent gene duplication, including these tandemly duplicated gene clusters. Some of these TE-affected genes are potentially involved in fatty acid biosynthesis and flower traits, implying that TE movements also promote genic innovation. Interestingly, the bursts of TE proliferation coincide with the uplift and glacial periods of the QTP, which is also reported in other QTP plants, like *C. himalaica* [45]. This further implies that active TE movements may also contribute to environmental adaptation after dramatic climate changes as duplicated gene copies derived from TEs provide bases for gene neofunctionalization and subfunctionalization [45].

With this high-quality genome, we characterized candidate genes involving the biosynthesis of flavonoids and terpenes. Previous studies have demonstrated the divergence of these secondary metabolites across *Paeonia* species with different flower color and scent [14, 15]. *Paeonia ludlowii* has rare pure yellow flowers different from those of other tree peony species, and contains the subsection Delavayanae-specific monoterpene linalool in flowers. Further studies on *P. ludlowii* and more peony resources will help disentangle the genetic mechanisms and transcriptional regulation patterns underlying the evolutionary divergence of these traits. Besides, the candidate genes involved in the pathway of fatty acid biosynthesis identified in *P. ludlowii* can be further investigated and utilized in breeding for human health, as *P. ludlowii* has a high content of unsaturated fatty acids.

In summary, we released a high-quality chromosome-level assembly of one wild tree peony, *P. ludlowii*, demonstrating substantial sequence divergence from other *Paeonia* genomes. Together with the genome-wide epigenomic data, we provide new insights into the evolution of the huge genome structure as well as the protein-coding genes. Our research will also contribute to breeding research on ornamental peonies and other applications, such as use as a woody oil crop.

## Materials and methods

### Sample preparation and whole-genome and transcriptome sequencing

Plants of *P. ludlowii* were grown in the national Tibetan Plateau crop germplasm garden at an altitude of ~3600 m. Young leaves from one plant were collected for DNA extraction and sequencing library preparation. One short-read paired-end library was constructed and sequenced with a read length of 150 bp on an Illumina Hiseq system. For PacBio HiFi sequencing, a high-molecular-weight DNA library was prepared using SMRTbell Express Template Prep Kit 2.0 and sequenced on a PacBio Sequel II platform. For RNA-seq, libraries were constructed from six different tissues, including roots, fruits, petals, buds, leaves, and branches, and sequenced on a NovaSeq 6000 platform. The fresh-year young roots and branches were sampled. The buds and young leaves were sampled at the flowering stage. The petals were sampled at the early flowering stage. The fruits were sampled 25, 50, 75, 100, 125, and 150 days after the end of the flowering stage, respectively, and mixed for RNA library construction. For Hi-C sequencing, young leaves of one plant were collected to extract high-quality genomic DNA samples. The samples were digested with 200 U DPN II restriction enzyme (Qiagen) for library construction. Hi-C libraries were controlled for quality and sequenced on an Illumina Novaseq platform with the model of 150 bp paired-end reads.

### Genome assembly

The genome size was estimated based on a 17-mer of 833 Gb Illumina paired-end reads. The *k*-mer was counted by Jellyfish v2.3.0 [46]. All HiFi reads of *P. ludlowii* were initially *de novo* assembled by the tools hifiasm v0.15.4 [21] with default parameters and HiCanu v2.1 [22] with ‘genomeSize = 10626 m’, respectively. Purge_dups v1.2.5 [47] was applied to analyze the haplotigs and overlaps in the assembly according to the read depth and remove the redundant sequences. To further improve the assembly quality of contigs from hifiasm, contigs from HiCanu were aligned to hifiasm contigs and used to fill the gaps using the tool quickmerge v0.3 [48] with a parameter setting of ‘-hco 5.0 -c 1.5 -l 889376 -ml 5000’. Then, we used BLASTN v2.13.0 with the parameter ‘-evalue 1e-10’ to remove contigs containing chloroplast genome sequences of *Paeonia jishanensis*, *P. delavayi*, and *P. qiui* [49]. Finally, the paired-end Hi-C reads were mapped to the contigs by HiC-Pro v3.1.0 [50]. ALLHiC v0.9.13 [51] was utilized to anchor contigs into five pseudochromosomes based on Hi-C read mappings. To refine the anchoring, Juicebox v1.11.08 [52] was used for manual correction.

We used three different methods to evaluate the genome assembly quality. First, BUSCO v5.3.0 [53] was used to evaluate genome completeness by searching the eudicots_odb10 database of 2326 genes. Second, HiFi reads, Illumina paired-end reads, and RNA-seq reads were mapped to the genome assembly for consistent assessment using minimap2 [54], Bowtie2 v2.4.5 [55], and HISAT2 v2.2.1 [56], respectively. Third, Merqury v1.3 [57] was applied to estimate base-level accuracy and completeness.

### Gene annotation

Gene prediction was based on three types of evidence: *ab initio* prediction, protein homology alignments, and RNA-seq read mapping. Augustus v3.3.1 [58], GlimmerHMM v3.0.4 [59], and SNAP v2006-07-28 [60] were used for *ab initio* gene model prediction. Exonerate v2.4.0 [61] was applied for aligning the protein sequences of *A. thaliana*, *K. fedtschenkoi*, and *Rhodiola crenulata* to the genome assembly. RNA-seq reads were aligned to the genome using HISAT2 v2.2.1 [56], and this was followed by reference-guided assembling by StringTie v2.1.4 [62]. Then, these results were integrated using EVidenceModeler v2012-06-25 [63]. To obtain a high-quality annotated gene set, we filtered the *ab initio* predictions to keep only those that were supported by at least two *ab initio* tools. After that, we removed genes overlapping with TEs with an overlap threshold of 30% of coding regions.

We used the tool INFERNAL v1.1.3 [64] with the database Rfam v14.6 [65] to annotate non-coding genes, including rRNAs, miRNAs, snRNAs, snoRNAs, and tRNAs. For lncRNA annotation, RNA-seq reads were assembled into transcripts using StringTie v2.1.4 [62], and the Perl script FEELnc_filter.pl of FEELnc [66] was utilized to remove the genes that overlapped with gene exons <200 bp in length. Then, these filtered transcripts were mapped to the protein sequences of the Swiss-Prot (http://www.gpmaw.com/html/swiss-prot.html) database by BLASTX v2.13.0 with an E-value of 1e−5 to remove strong hits.

Gene functional annotation was performed by BLASTP alignment against databases of RefSeq non-redundant proteins (https://www.ncbi.nlm.nih.gov/refseq/about/nonredundantproteins/) and Swiss-Prot (http://www.gpmaw.com/html/swiss-prot.html) with ‘-evalue 1e-5’. The motif and domain information were annotated by integrating results from Pfam [67], CDD [68], SMART [69], and PANTHER [70] with InterProscan v5.55–88.0 [71]. The GO term was added using eggNOG v2.1.7 [72]. KEGG annotation was performed by the KEGG Automatic Annotation Server (KASS, https://www.genome.jp/tools/kaas/). Transcription factors were identified using PlanTFDB v5.0 [73].

### Genome alignment and structural variation identification

The current whole-genome alignment tools fail to directly align chromosomes >2 147 483 647 bp. To do homologous chromosome alignments between *P. ludlowii* and *P. ostii*, each chromosome was split into two equal parts. Each part was aligned separately using MUMmer v4.0.0rc1 [23] with the parameters ‘--mum -D 5’. The results were filtered with ‘delta-filter -1 -i 80 -l 200’. Then, the sequence variations, including SNPs, indels, and SVs, were identified by SyRI v1.6.3 [74] with default settings. Plotsr v0.5.4 [75] was used to visualize SV results.

The genome assembly of *P. suffruticosa* was not at the chromosome level. In order to quickly obtain the difference between it and *P. ludlowii* and *P. ostii*, two methods (assembly level, read level) were used for alignment. (i) We randomly selected 1000 contig sequences of the genome of *P. suffruticosa*, and mapped them to the genomes of *P. ludlowii* and *P. ostii* with minimap2. Based on the alignments, the matching rate was calculated. (ii) Minimap2 was used to map the reads of *P. suffruticosa* to the genomes of *P. ludlowii* and *P. ostii*, and then the matching rate was calculated.

### Repeat annotation and analyses

Repeats of *P. ludlowii* were annotated by integrating two *de novo* methods. In brief, EDTA v2.0.0 [76] and RepeatModeler v2.0.1 [77] were used to build a *de novo* repeat library. Then, we merged the two libraries and removed the redundant sequences using the tool cd-hit v4.8.1 [78] to get an integrated repeat library. Finally, RepeatMasker v4.1.1 [79] was applied for repeat annotation and genomic masking with this integrated library. TEsorter v1.3 [80] was used to classify the TEs. Intact LTRs were identified based on the EDTA pipeline, and solo LTRs were identified based on the method of Wan *et al.* [81].

To calculate the insertion time of LTR retrotransposons, we first used MAFFT v7.490 [82] to align the 5′-LTR and 3′-LTR at both ends of each intact LTR, then calculated the distance using the dismat tool in the EMBOSS package (http://emboss.sourceforge.net). The insertion time was estimated based on the formula *T* = *K*/2*r*, where *K* is the genetic distance between LTRs and *r* is the rate of nucleotide substitution. We set *r* (7 × 10^−9^) as the rate of nucleotide substitution in *A. thaliana* [83]. Tandem repeats were predicted using TRF v4.09 [84]. Then, the results were filtered and clustered to find the candidate centromeric tandem repeat unit [30, 85].

### Phylogenetic analyses

To explore the evolutionary position of *P. ludlowii*, we applied both concatenated and coalescence strategies for phylogenetic analysis. A total of 22 representative plant genomes were selected, including *A. thaliana*, *Theobroma cacao*, *Citrus sinensis*, *Euscaphis japonica*, *Averrhoa carambola*, *Glycine max*, *Carya illinoinensis*, *Prunus persica*, *Solanum tuberosum*, *Olea europaea*, *Ophiorrhiza pumila*, *Rhododendron simsii*, *Apium graveolens*, *Lactuca sativa*, *Vitis vinifera*, *P. ludlowii*, *C. japonicum*, *K. fedtschenkoi*, *R. crenulata*, *O. sativa*, *A. coerulea*, and *B. sinica*. Among them, *O. sativa*, *A. coerulea*, and *B. sinica* were selected as the outgroup. For genes with multiple alternative isoforms, the longest was preserved. Protein sequences from these genomes were aligned all-versus-all. Orthologous groups were identified by Orthofinder v2.5.4 [86] based on these protein alignments. These groups were further classified into strict single-copy genes (SSGs) and low-copy-number genes (LCNGs) based on the number of orthologous genes of each species in each group. We further divided the LCNG groups into LCNG1, LCNG2, LCNG3, LCNG4, and LCNG5, of which at most one, two, three, four, and five species had multiple orthologous genes.

For each gene set, multiple sequence alignments of protein sequences were performed by MUSCLE v5.1 [87], and further converted to nucleotide alignments by the tool PAL2NAL v14 [88], followed by gap removal with the tool trimAl v1.4.rev22 [89] under ‘-automated1’ mode. The maximum likelihood tree for each gene set was built by IQ-TREE2 v2.2.0 [24] with ‘-m MFP -bb 1000’. All gene trees were then merged into a species tree by ASTRAL v5.7.8 [90] and ASTRAL-Pro v1.4.1.3 [91] with the multi-species coalescent mode. The species divergence time was calculated by MCMCTree v4.10.0 in the PAML package [92]. The species calibration time was searched at TimeTree (https://www.timetree.org), including *S. tuberosum* and *O. europaea* (0.724–1.049 Mya), *L. sativa* and *A. graveolens* (0.756–0.904 Mya), *K. fedtschenkoi* and *R. crenulata* (0.396–0.452 Mya), *P. persica* and *C. illinoinensis* (0.89–1.059 Mya), *A. thaliana* and *T. cacao* (0.83–0.931 Mya), *C. sinensis* and *T. cacao* (0.9–0.999 Mya), and *A. carambola* and *T. cacao* (1.02–1.138 Mya). The species tree inconsistency analysis was performed by DiscoVista v1.0 [25] software on the topological structure of all gene set species trees. Finally, the Interactive Tree Of Life (iTOL) [93] was applied for tree visualization.

Gene family expansion and contraction analyses were conducted by CAFÉ v5 [94]. The significantly expanded and contracted gene families were calculated under the *P*-value cutoff of 0.1.

### Identification of whole-genome duplication event

First, in order to search for paralogous genes within *A. coerulea*, *B. sinica*, *C. japonicum*, *V. vinifera*, and *P. ludlowii*, as well as orthologous genes between *P. ludlowii* and *A. coerulea*, *B. sinica*, *C. japonicum*, and *V. vinifera*, all-versus-all alignment of protein sequences was conducted by BLASTP v2.13.0 [95]. Subsequently, we utilized the WGDI tool v0.6.1 [96] with improved collinearity (−icl) mode to identify collinear blocks. The synonymous substitution (*K*_s_) median of each block was calculated by WGDI (−ks), and the *K*_s_ plot was fitted based on a Gaussian distribution by WGDI (−kf). NGenomeSyn v1.4.0 [97] was used to visualize microcollinearity between chromosomes. Based on the core eudicot ancestor karyotype provided by WGDI, the karyotype compositions of *A. thaliana*, *Populus trichocarpa*, *P. ludlowii*, *C. japonicum*, and *V. vinifera* were predicted using WGDI (−km).

### DNA methylation sequencing and data analyses

DNA was extracted from the young leaves of one *P. ludlowii* plant and treated with bisulfite using the Scale Methyl-DNA Lib Prep Kit for Illumina. The library was constructed by Novogene Corporation (Beijing, China) and sequenced on an Illumina Novaseq platform.

Because the chromosome size of peony exceeds the limit of all current state-of-the-art BS-seq read aligners, we split each chromosome into two equal parts for read mapping and methylated site calling. The results of split chromosomes were merged at the final stage. All high-quality WGBS reads from two replicated samples were mapped to the *P. ludlowii* assembly using Bismark v0.22.3 [98]. The PCR duplication was removed using the deduplicate_bismark program, and only uniquely mapped reads were retained. Then, the bismark_methylation_extractor program was used to calculate the number of methylated cytosines with parameters ‘-p --comprehensive --no_overlap --CX --bedGraph --counts --parallel 10 --buffer_size 30G --cytosine_report’. Then, the methylation levels of CG, CHG, and CHH were calculated using methyGff v1.0 from the BatMeth2 [99] software package.

### Gene duplication analyses

A modified DupGen_finder [100] pipeline was used to identify gene duplication. In brief, all-versus-all BLASTP was used to search for potential homologous gene pairs, and WGDI (−icl) was used to identify WGD-derived duplication gene pairs. Then, we used DupGen_finder to identify DSD, PD, TD, and TRD duplicated gene pairs.

To calculate *K*_s_ values of duplicated gene pairs, we first performed protein sequence alignments by MAFFT and transformed the alignments into the nucleotide level using ParaAT v1.0 [101]. Then, the *K*_s_ value was calculated by KaKs_Calculator v2.0 [102] based on the nucleotide-level alignments.

### Gene family annotation

To annotate the fatty acid desaturase (FAD) gene family, the genes involved in the fatty acid pathway in *Arabidopsis* were used to search candidate genes in *P. ludlowii* by BLASTP v2.13.0 with the following cutoffs: E-value <0.05, query coverage >50%, and identity >50%. Besides, FAD genes were searched using HMMER v3.3.1 [103], based on domains of PF00487 and PF03405 from the Pfam database. Then, we manually removed redundant hits based on the length of the protein sequence and obtained the final FAD gene set. To identify potential genes for terpenoid biosynthesis in *P. ludlowii*, BLASTP v2.13.0 (E-value <1e−5, identity >50%, and coverage >50%) was used for alignment against homologous protein sequences in *A. thaliana*. In addition, for the TPS gene, we also utilized hmmsearch in HMMER v3.3.1 based on the PF01397 and PF03936 Pfam domains, then merged these two results and manually removed redundant hits. To identify the candidate genes in the pathway of flavonoid biosynthesis, structured genes in *Arabidopsis* were used as queries for homologous gene searching in *P. ludlowii*, as described above.

## Supplementary Material

Web_Material_uhad241

## Data Availability

All raw sequencing data and assembly sequences have been deposited at the National Genomics Data Center (https://ngdc.cncb.ac.cn) under BioProject accession number PRJCA016714. The assembly sequences and annotation files have been deposited in Figshare (https://doi.org/10.6084/m9.figshare.23537670).
