# Supplementary material for: High-quality assembly and methylome of a Tibetan wild tree peony genome (Paeonia ludlowii) reveal the evolution of giant genome architecture
Source: Hortic Res. 2023 Nov 10;10(12):uhad241. doi: 10.1093/hr/uhad241 (PMC10753165; doi:10.1093/hr/uhad241)
Supplement: Web_Material_uhad241 [file web_material_uhad241.zip › Supplementary-Figures.pdf]

Supplementary Figures

Figure S1. The flower of *P. ludlowii*.

Figure S2. K-mer (k = 17) based prediction of genome size for *P. ludlowii*.

Figure S3. The chromatin interaction map of the *P. ludlowii* genome based on Hi-C data.

Figure S4. Differences in intron sizes between homologous genes of *P. ludlowii* and *P. ostii.*

Figure S5. Phylogeny result based on the LCNG3 dataset.

Figure S6. Phylogeny result based on the SSG dataset.

Figure S7. Phylogeny result based on the LCNG1 dataset.

Figure S8. Phylogeny result based on the LCNG2 dataset.

Figure S9. Phylogeny result based on the LCNG4 and LCNG5 dataset.

Figure S10. Visualization of phylogenetic inconsistency with the tool DiscoVista.

Figure S11. Divergence time of 20 representative plant species.

Figure S12. Syntenic Ks dotplot within and between species.

Figure S13. Chromosome karyotype comparisons between *P. ludlowii* and grape.

Figure S14. Ratios between solo LTR and intact LTR across different plant genomes.

Figure S15. Ks distribution of duplicated gene pairs.

Figure S16. Expression levels of genes with or without TE-inserted introns.

Figure S17. Counts of genes involved in DNA methylation and demethylation pathways in Saxifragales and Vitales genomes.

Figure S18. Gene number of the FAD gene family identified in *P. ludlowii* and other six plant genomes.

Figure S19. Expression, TE insertion, and DNA methylation of genes potentially involving the biosynthesis of fatty acids.

Figure S20. Distribution of FAD genes on *P. ludlowii* chromosomes.

Figure S21 Expression, TE insertion, and DNA methylation of genes potentially involving flower color pathway.

Figure S22. Gene number of the TPS gene family identified in *P. ludlowii* and other nine plant genomes.

Figure S23. Expression, DNA methylation, TE insertion, TPS type, duplication type, and intron-to-gene length ratio of TPS gene family.

Figure S24. Micro-synteny of one tandem TPS-b gene cluster among *P. ludlowii*, *P. ostii*, and grape (*V. vinifera*) genomes.

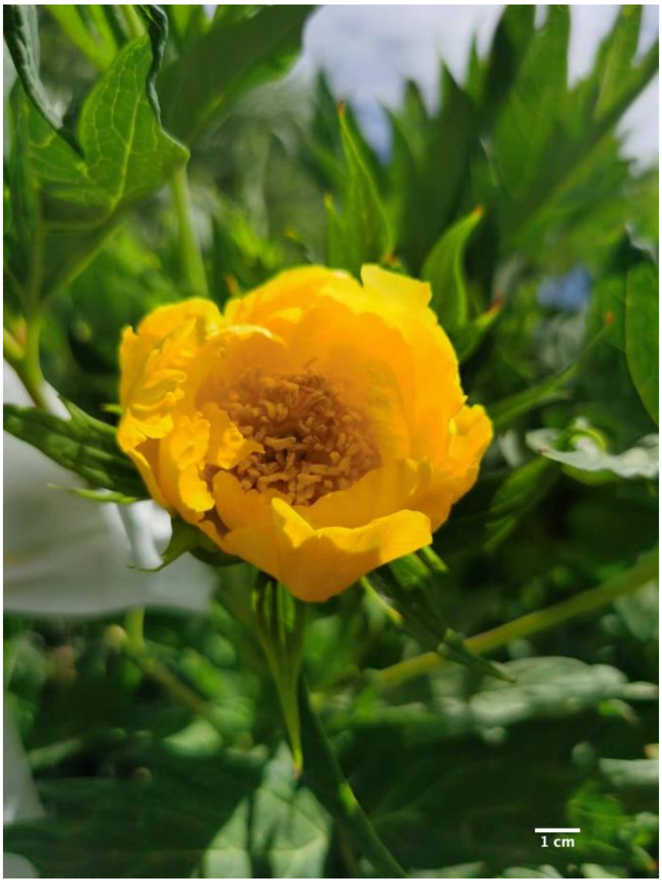

Figure S1. The flower of *P. ludlowii*.

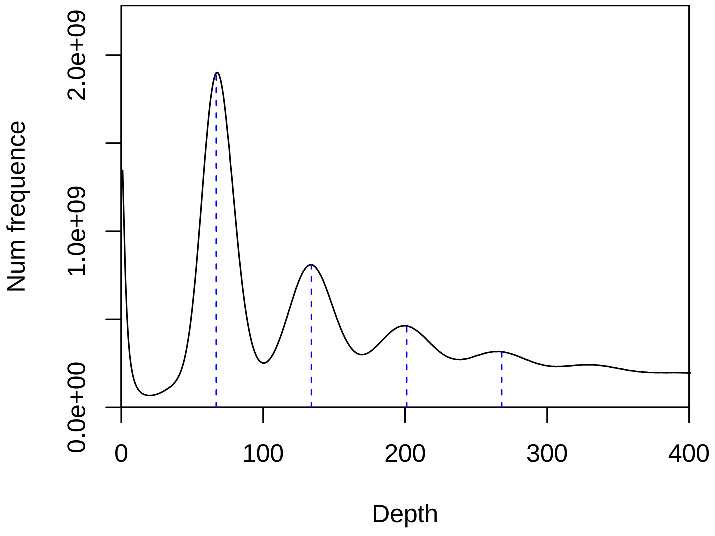

Figure S2. K-mer (k = 17) based prediction of genome size for *P. ludlowii*. The curve shows the frequency distribution of the number of k-mers. The x-axis represents k-mer depth. The blue dotted line represents the depth of k-mer peak, and the main peak depth is about 67.

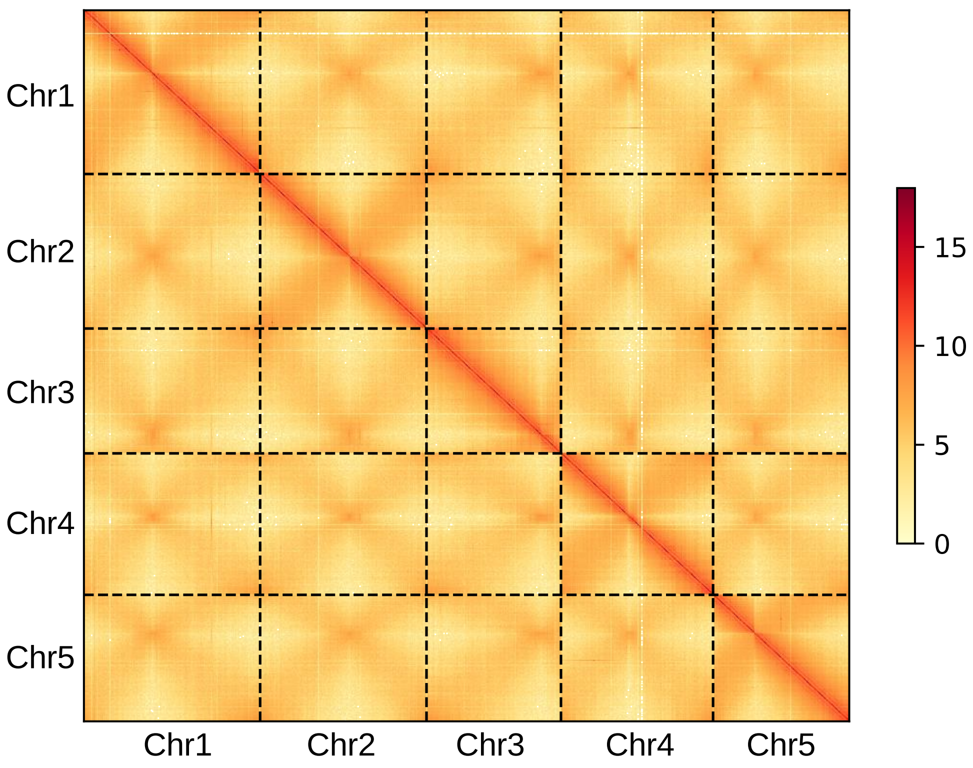

Figure S3. The chromatin interaction map of the *P. ludlowii* genome based on Hi-C data.

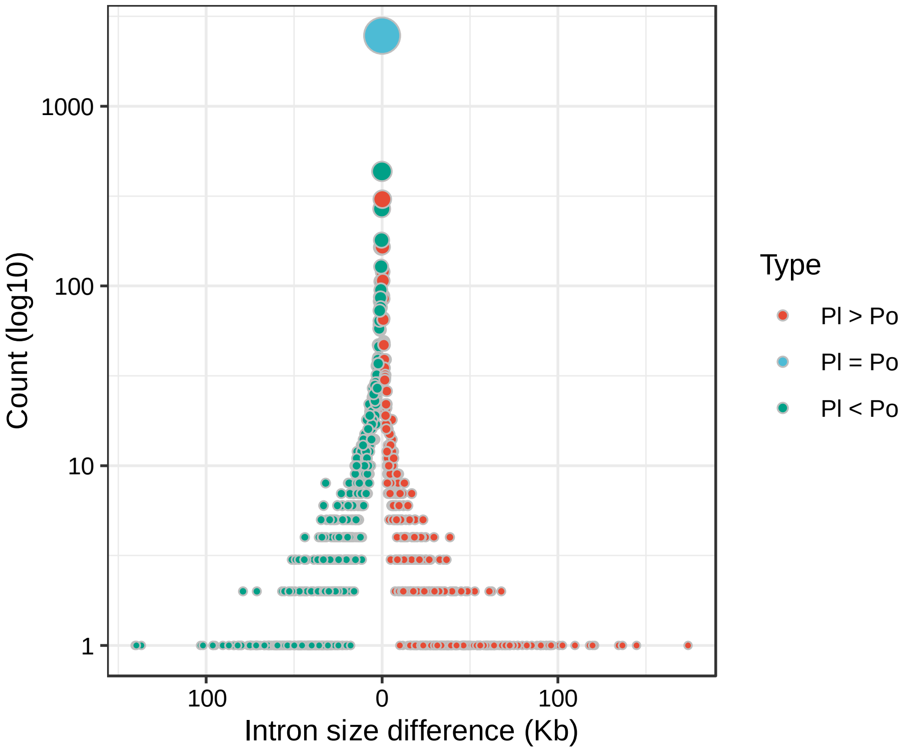

Figure S4. Differences in intron sizes between homologous genes of *P. ludlowii* and *P. ostii*. The x-axis represents the intron size difference between *P. ludlowii* and *P. ostii*, y-axis represents the number of genes in the corresponding intron size interval. The size of the points represents the number of genes, and the colors of points represent intron size relationships: Pl > Po, Pl = Po, Pl < Po, where Pl refers to *P. ludlowii* and Po refers to *P. ostii*.

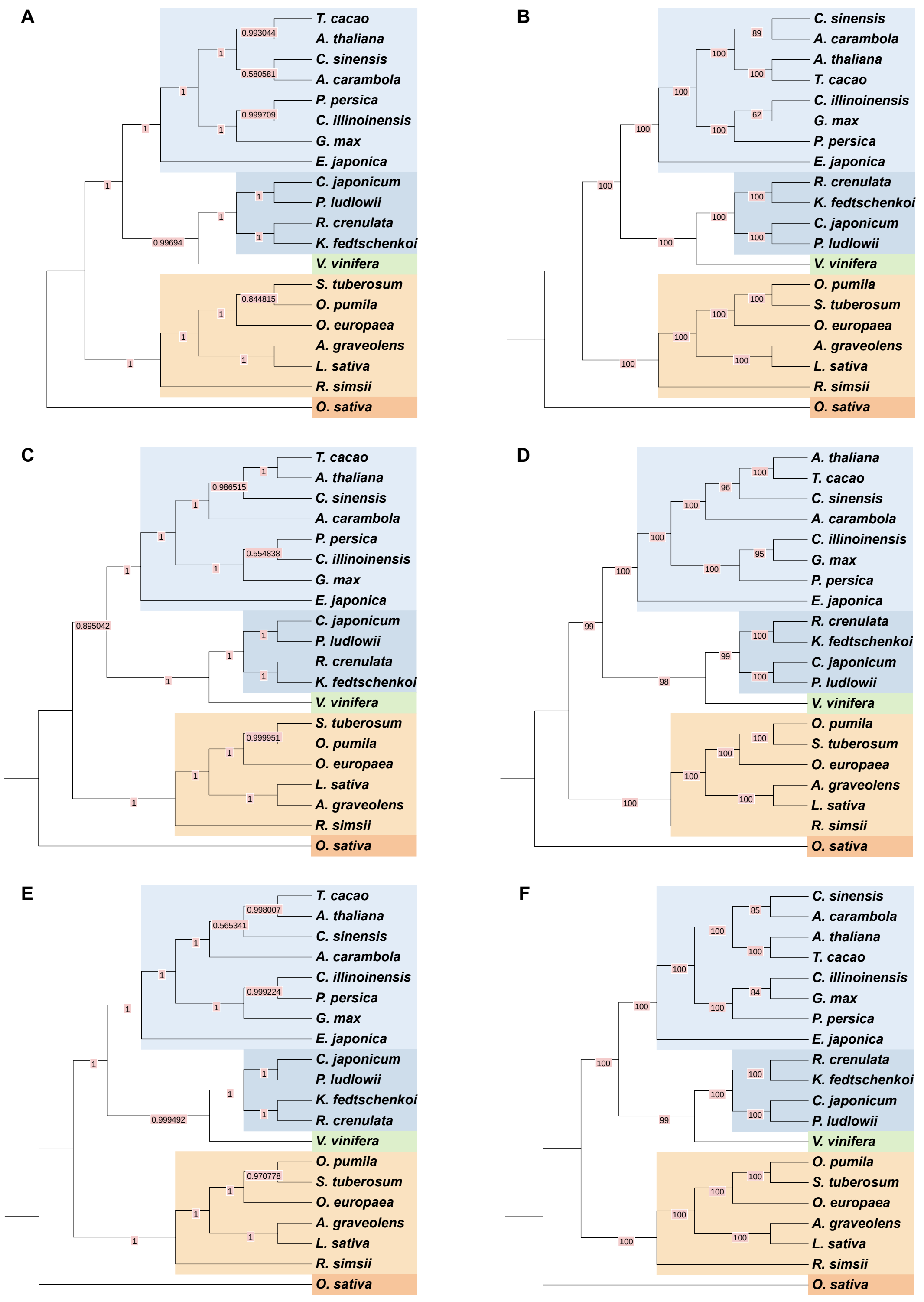

Figure S5. Phylogeny result based on the LCNG3 dataset. (A, B) The protein result based on coalescence and concatenated method, respectively. **(C, D)** The DNA result based on coalescence and concatenated method, respectively. **(E, F)** The codon 1&&2 result based on coalescence and concatenated method, respectively. The number on the branch represents bootstrap values (concatenated) and posterior probabilities (coalescence); different background colors represent different species class.

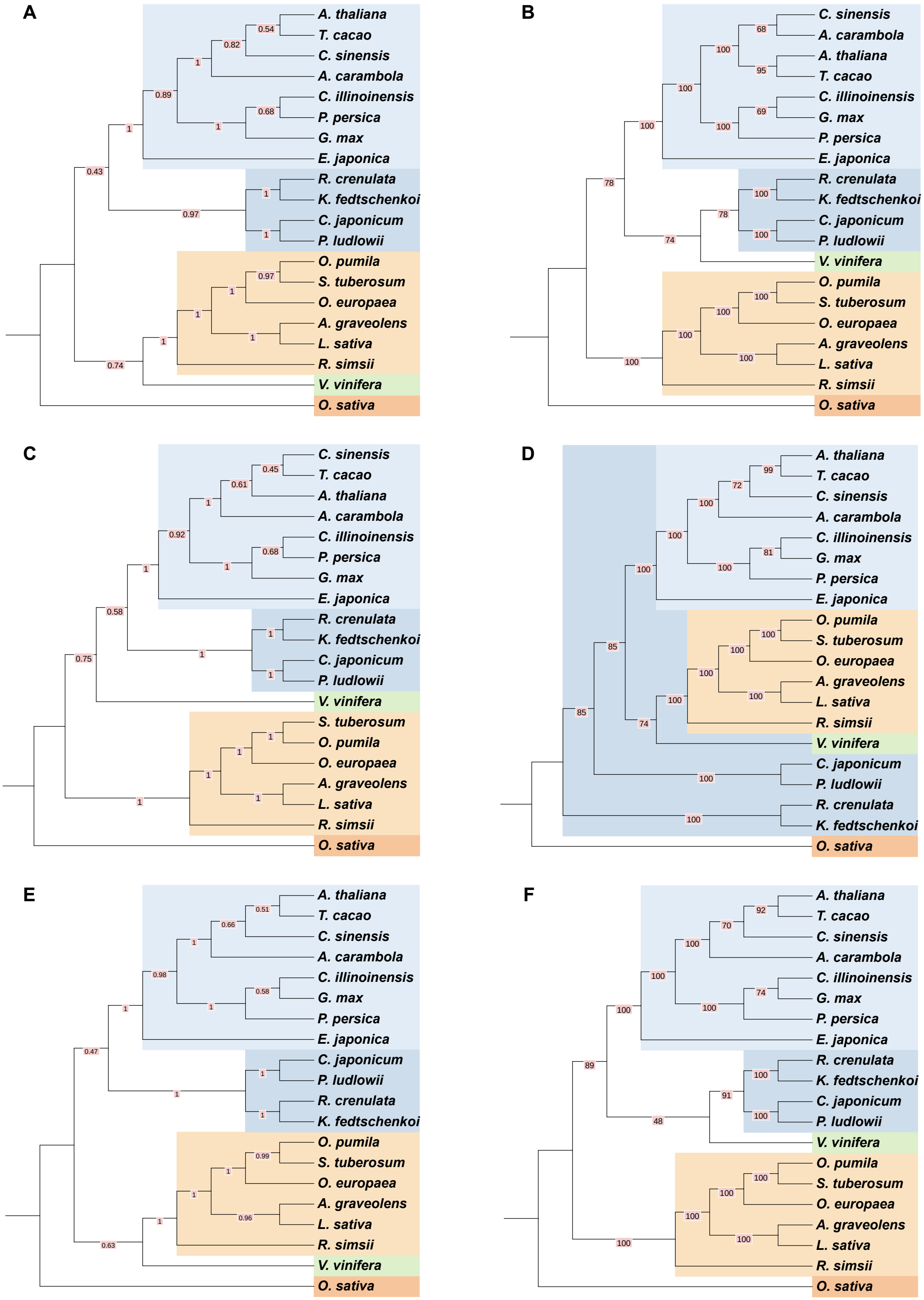

Figure S6. Phylogeny result based on the SSG dataset. (A, B) The protein result based on coalescence and concatenated method, respectively. **(C, D)** The DNA result based on coalescence and concatenated method, respectively. **(E, F)** The codon 1&&2 result based on coalescence and concatenated method, respectively. The number on the branch represents bootstrap values (concatenated) and posterior probabilities (coalescence); different background colors represent different species class.

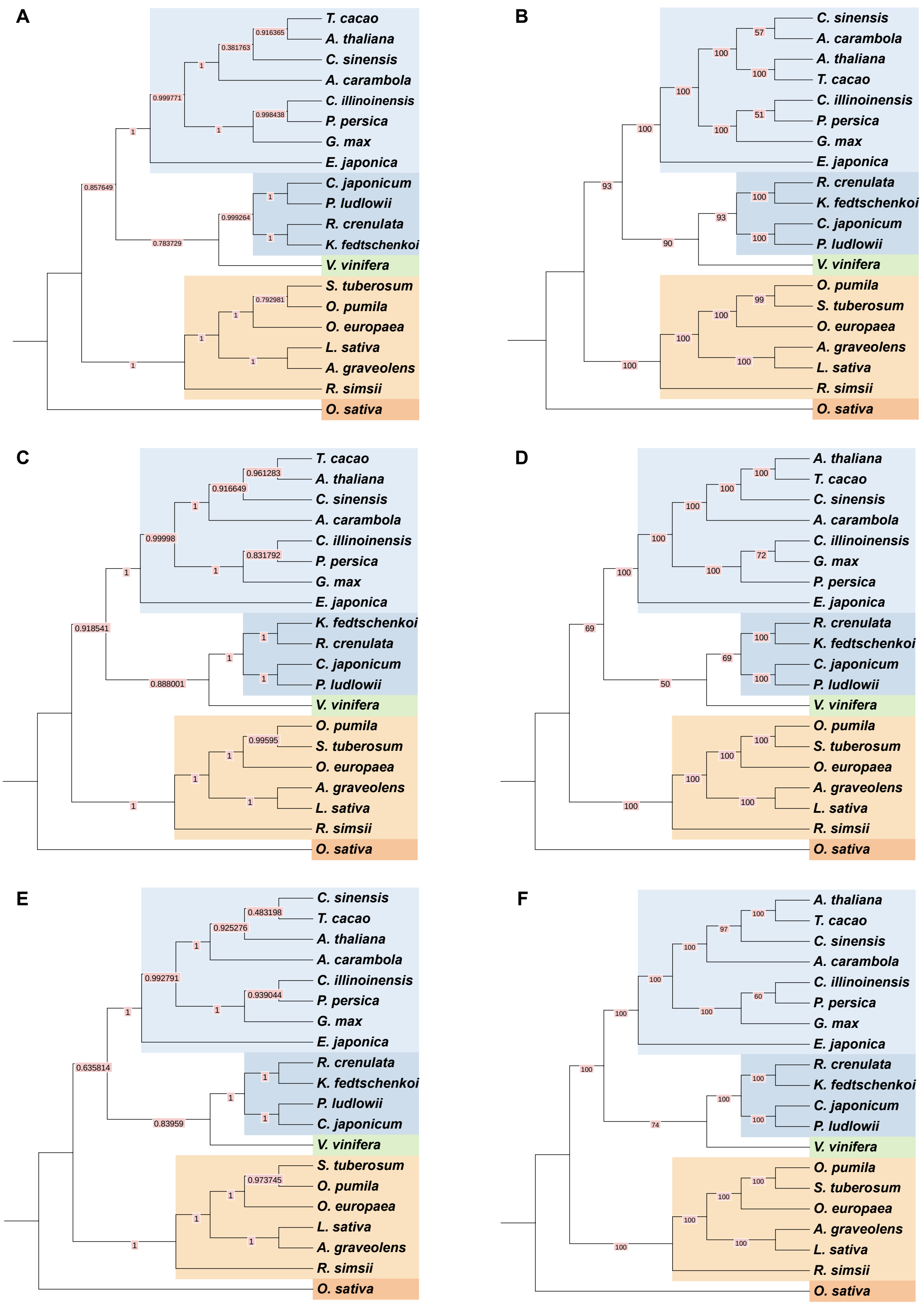

Figure S7. Phylogeny result based on the LCNG1 dataset. (A, B) The protein result based on coalescence and concatenated method, respectively. **(C, D)** The DNA result based on coalescence and concatenated method, respectively. **(E, F)** The codon 1&&2 result based on coalescence and concatenated method, respectively. The number on the branch represents bootstrap values (concatenated) and posterior probabilities (coalescence); different background colors represent different species class.

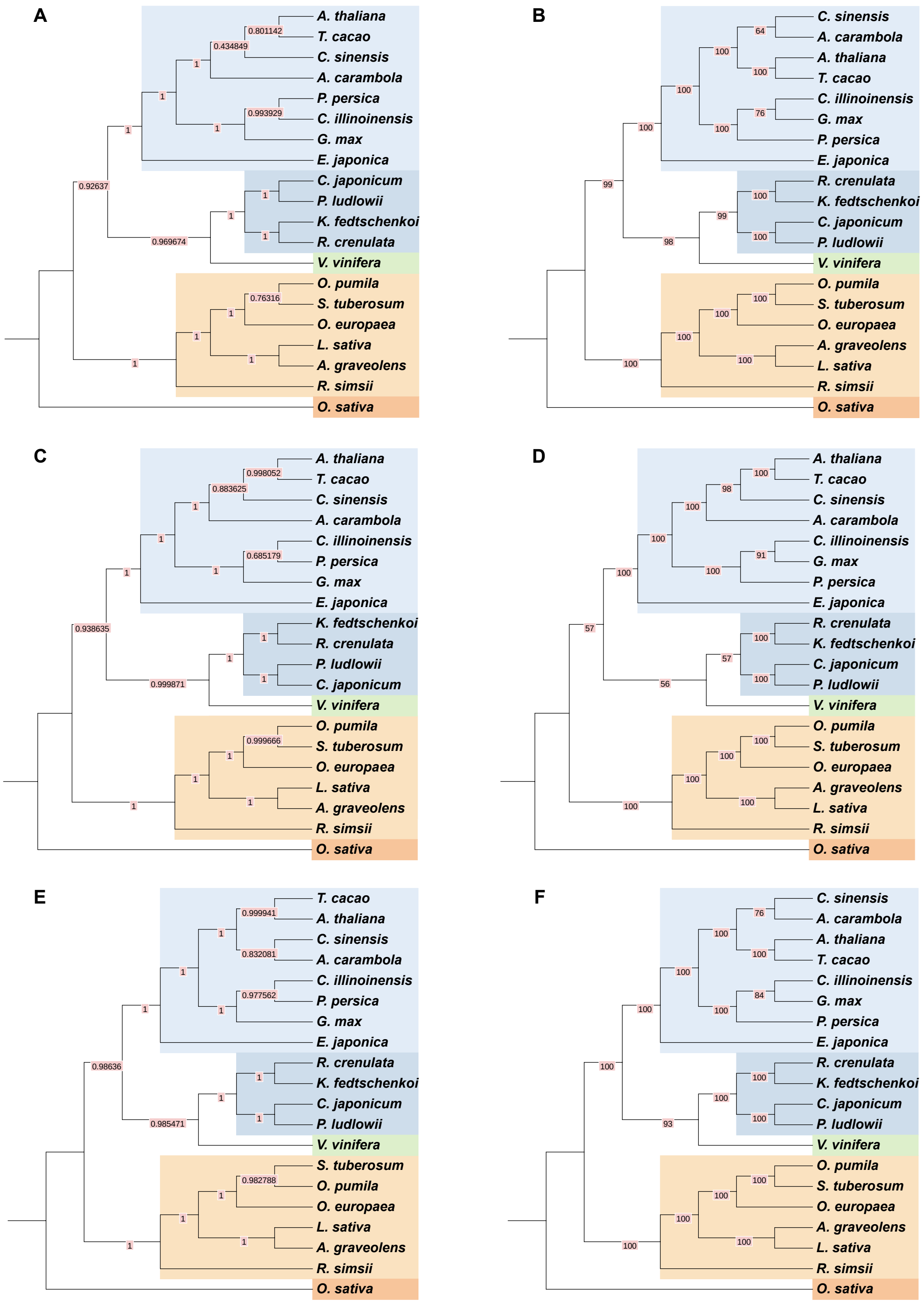

Figure S8. Phylogeny result based on the LCNG2 dataset. (A, B) The protein result based on coalescence and concatenated method, respectively. **(C, D)** The DNA result based on coalescence and concatenated method, respectively. **(E, F)** The codon 1&&2 result based on coalescence and concatenated method, respectively. The number on the branch represents bootstrap values (concatenated) and posterior probabilities (coalescence); different background colors represent different species class.

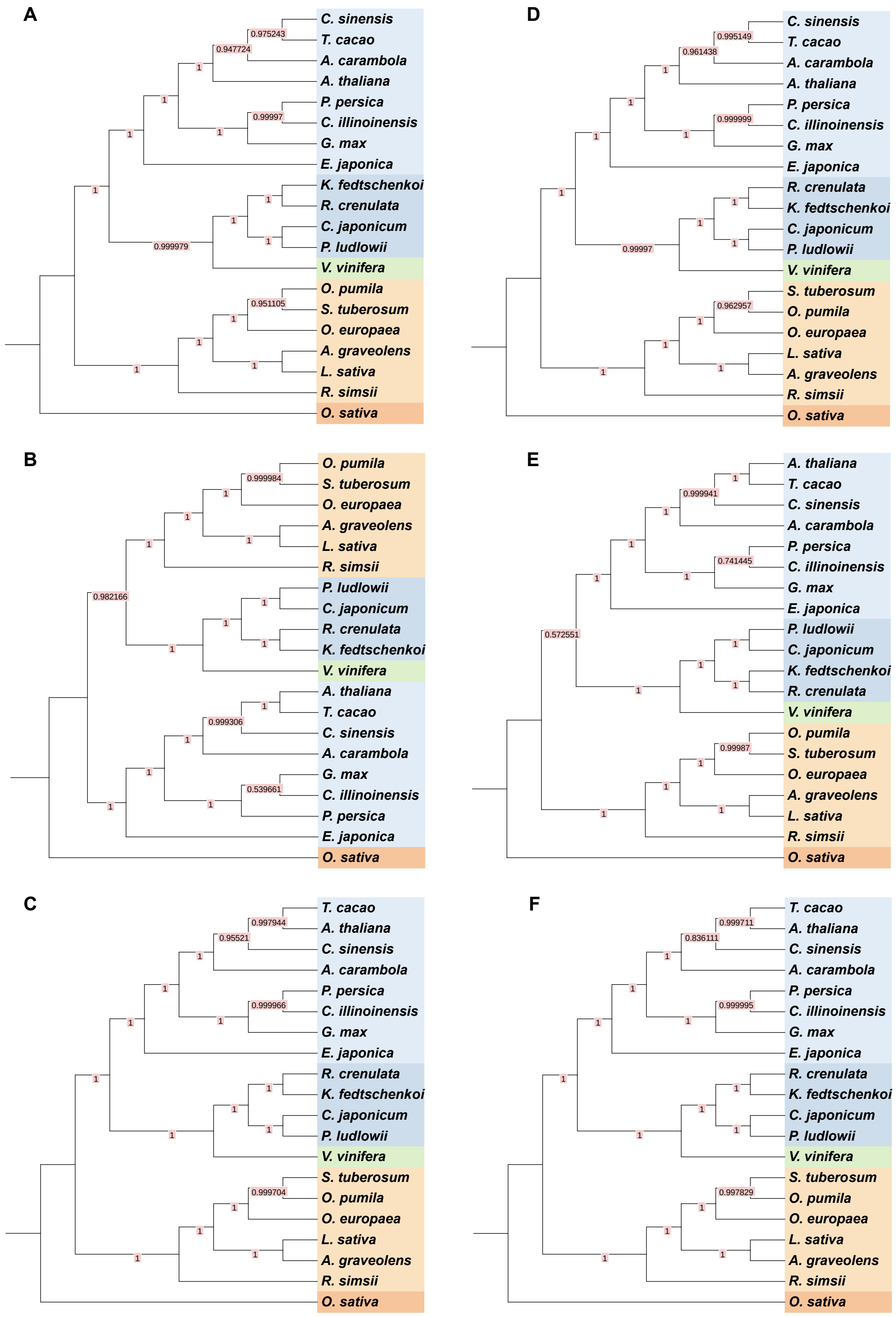

Figure S9. Phylogeny result based on the LCNG4 and LCNG5 dataset. (A, B, C) The protein, DNA, codon 1&&2 result of LCNG4 based on coalescence method. **(D, E, F)** The protein, DNA, codon 1&&2 result of LCNG5 based on coalescence method. The number on the branch represents bootstrap values (concatenated) and posterior probabilities (coalescence); different background colors represent different species class.

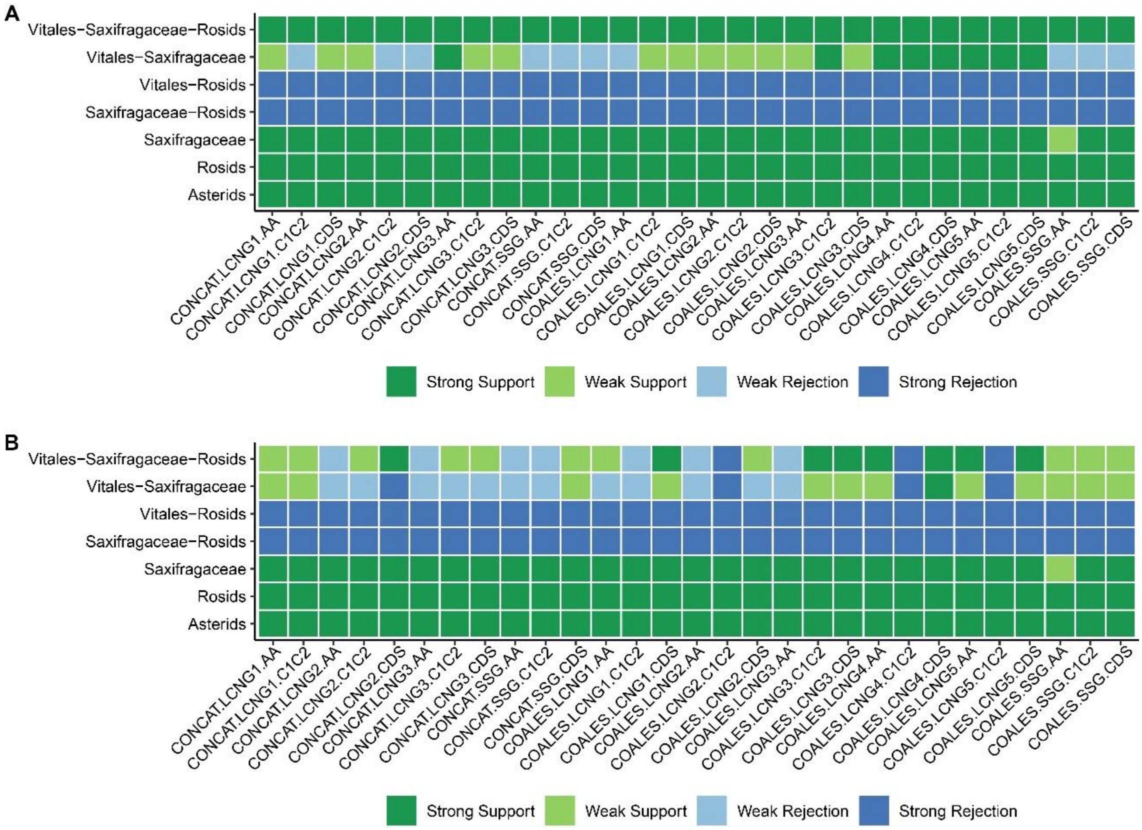

Figure S10. Visualization of phylogenetic inconsistency with the tool DiscoVista. (A) *Aquilegia coerulea* as outgroup. **(B)** *Buxus sinica* as outgroup.

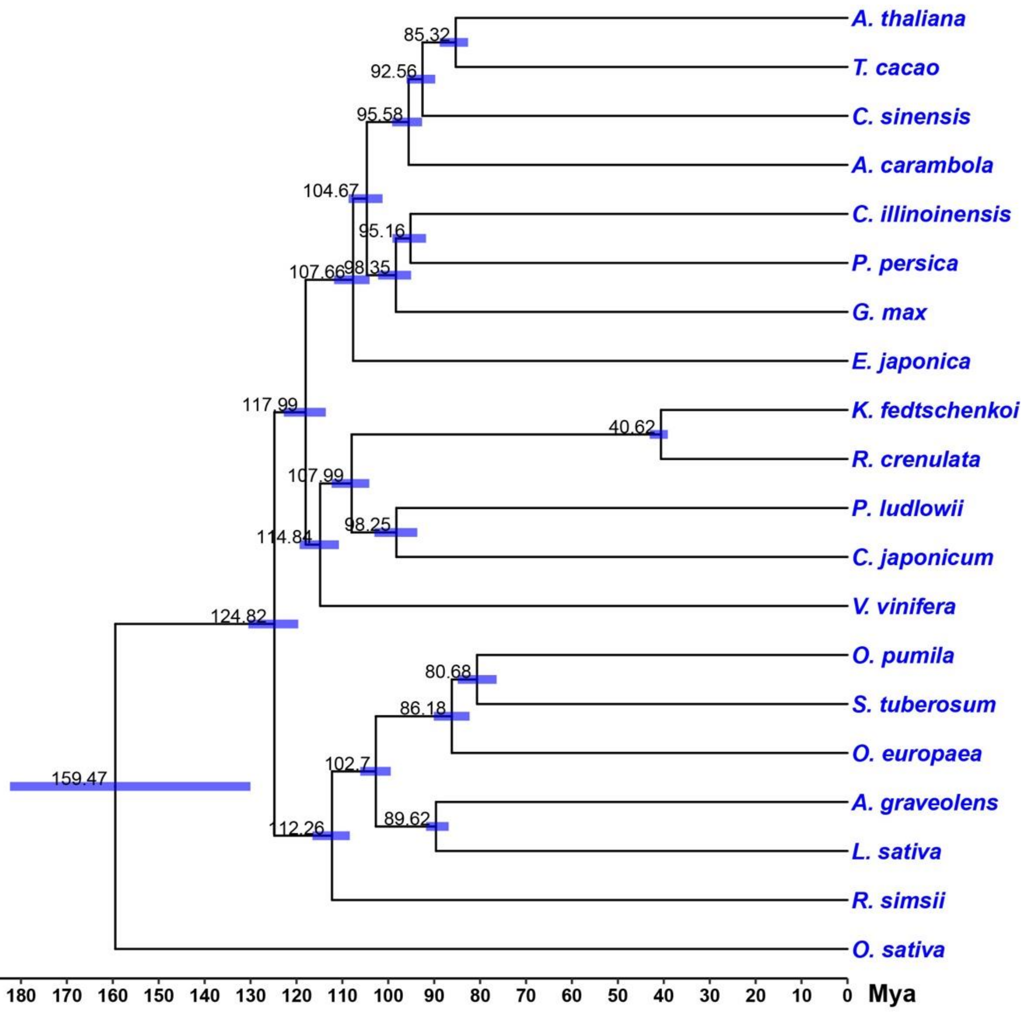

Figure S11. Divergence time of 20 representative plant species. Divergence time estimates for clades are shown at each node, with the 95% confidence interval denoted by blue bars. Mya: million years ago.

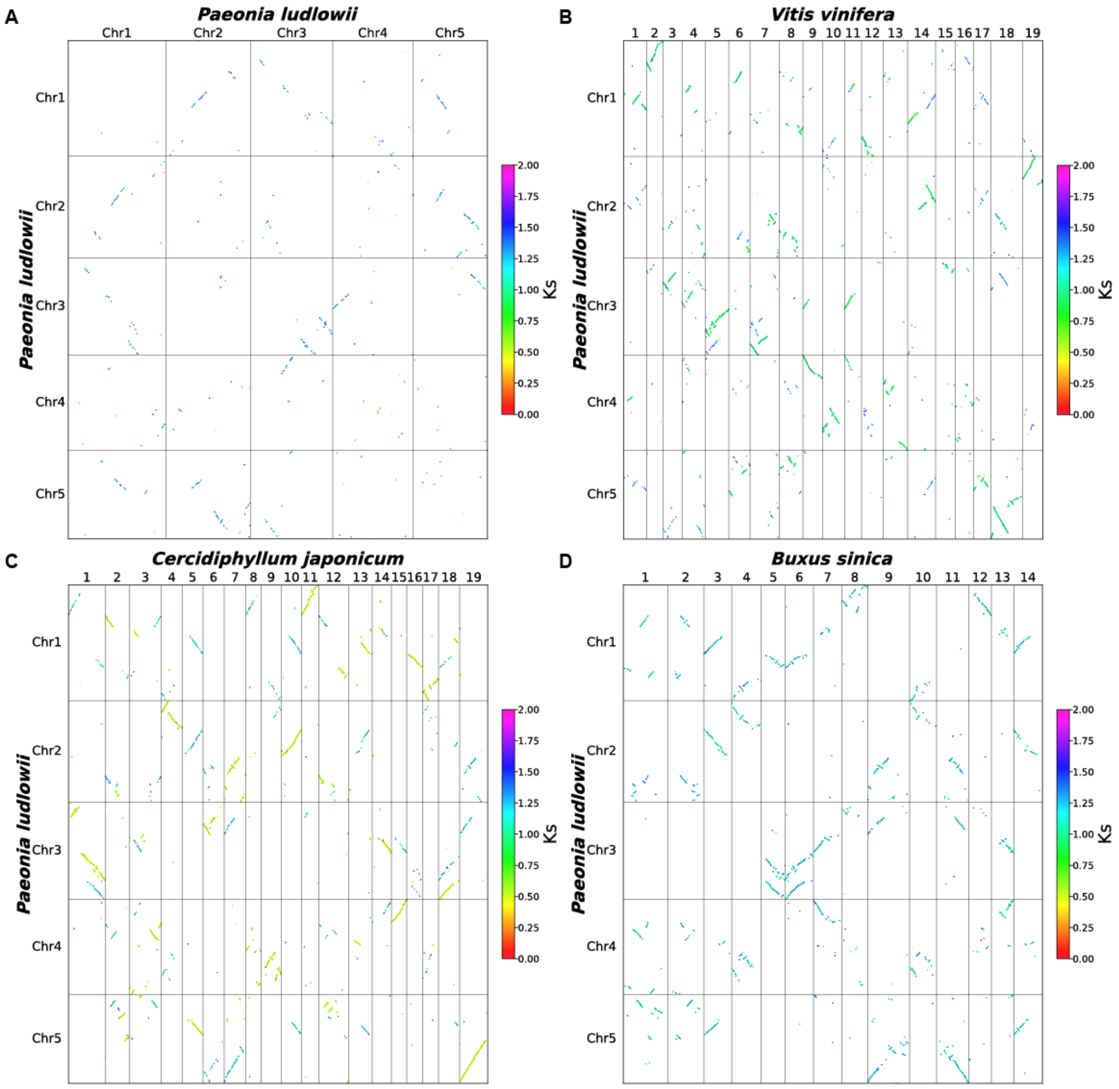

Figure S12. Syntenic *Ks* dotplot within and between species. (A) *Ks* dotplot within *P. ludlowii*. **(B, C, D)** *Ks* dotplot between *P. ludlowii* and *V. vinifera*, *C. japonicum*, and *B. sinica*. The color of the dots represents different *Ks* values.

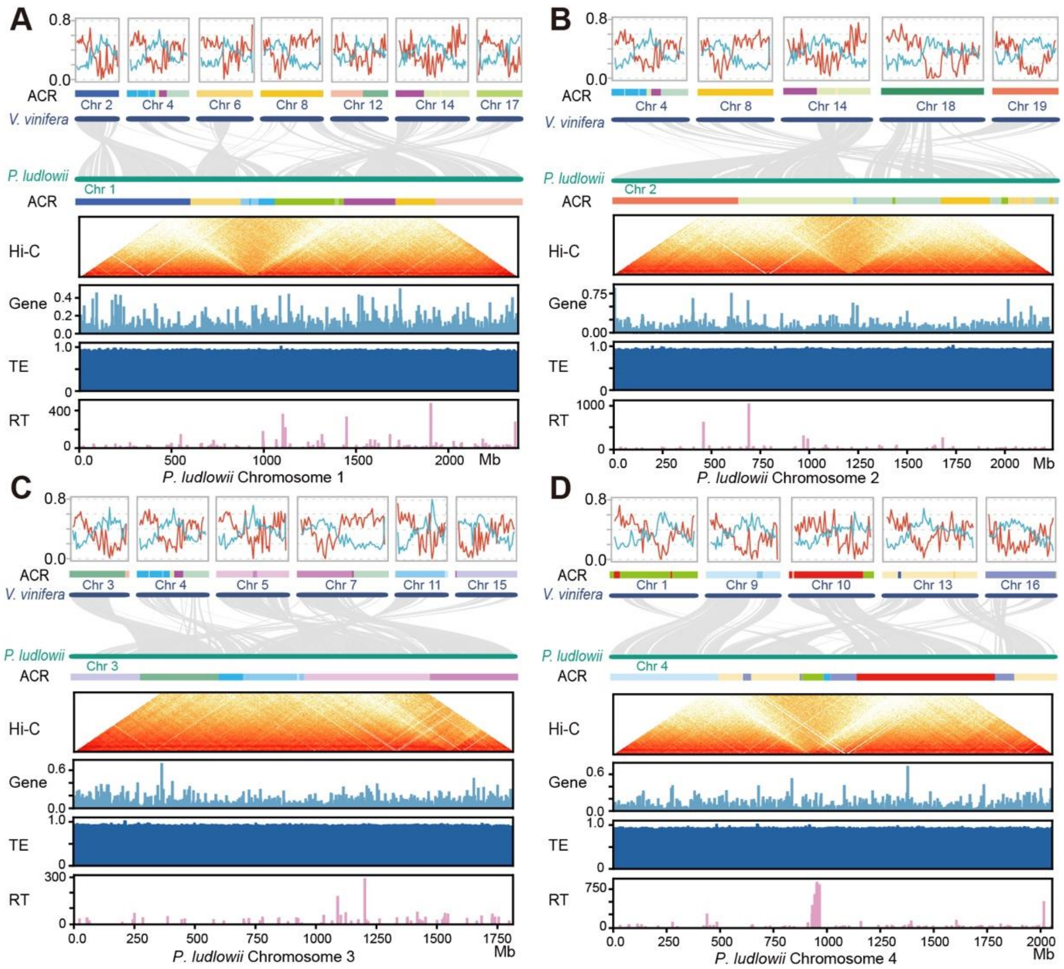

Figure S13. Chromosome karyotype comparisons between *P. ludlowii* and grape. (A) From top to bottom, the figure displays gene (red line) and TE (light blue line) density along chromosomes as well as composition of ancestral chromosome karyotype of grape genome, syntenic relationship with chromosome 1 of *P. ludlowii*; and composition of ancestral chromosome karyotype, Hi-C contact map, gene number, TE number and TR (tandem repeat) number of *P. ludlowii* chromosome 1. (B-D) Comparisons of *P. ludlowii* chromosome 2, 3, and 4 as (A).

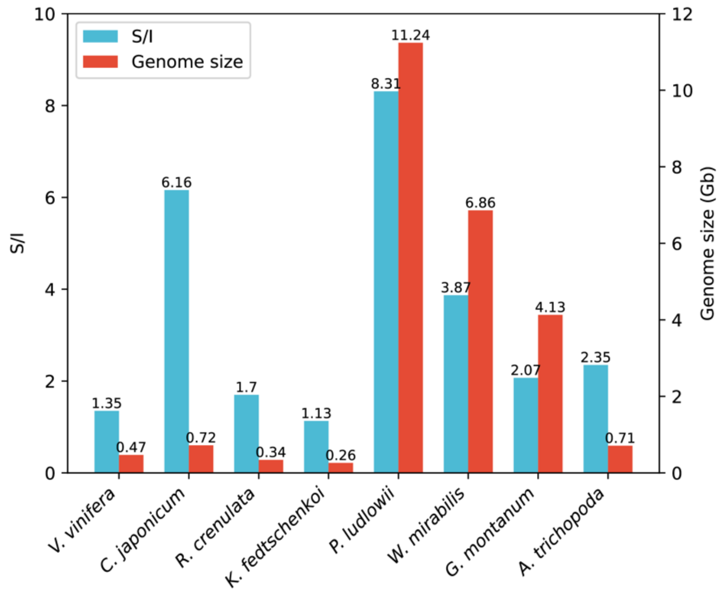

Figure S14. Ratios between solo LTR and intact LTR across different plant genomes. S/I: the ratio of solo LTR to intact LTR; *V. vinifera*: *Vitis vinifera*; *C. japonicum*: *Cercidiphyllum japonicum*; *R. crenulata*: *Rhodiola crenulata*; *K. fedtschenkoi*: *Kalanchoë fedtschenkoi*; *P. ludlowii: Paeonia ludlowii*; *W. mirabilis*: *Welwitschia mirabilis*; *G. montanum*: *Gnetum montanum*; *A. trichopoda*: *Amborella trichopoda*. The results of *W. mirabilis*, *G. montanum*, *and A. trichopoda* come from Wan *et al.* [1].

.

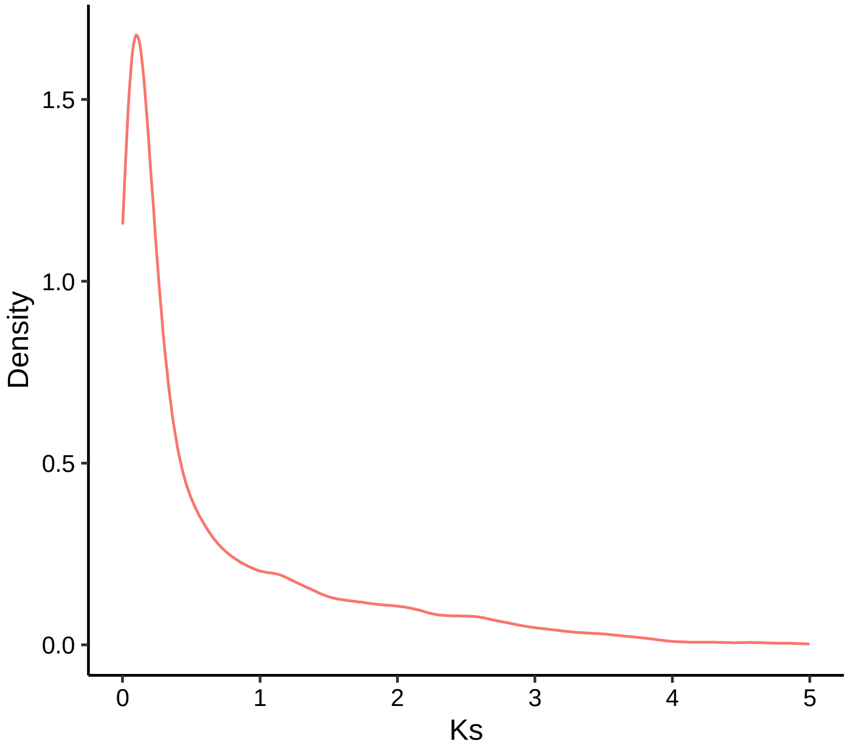

Figure S15. *Ks* distribution of duplicated gene pairs.

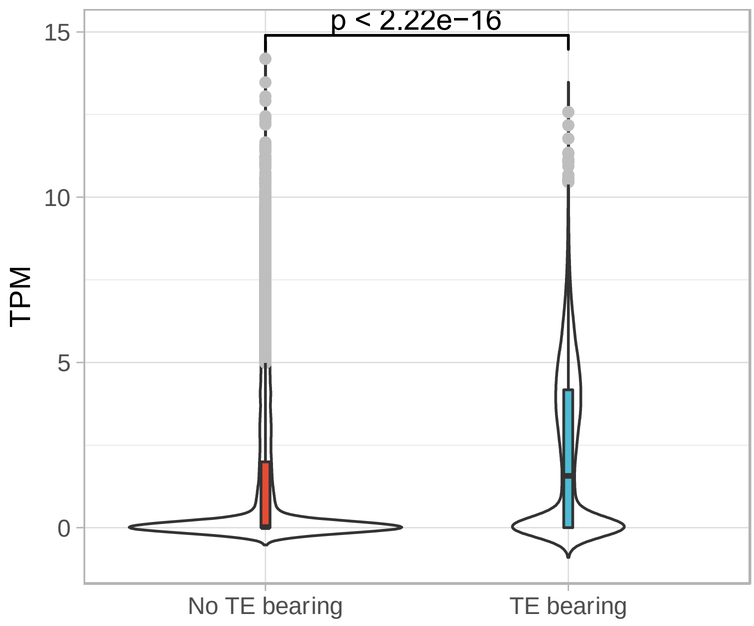

Figure S16. Expression levels of genes with or without TE-inserted introns.

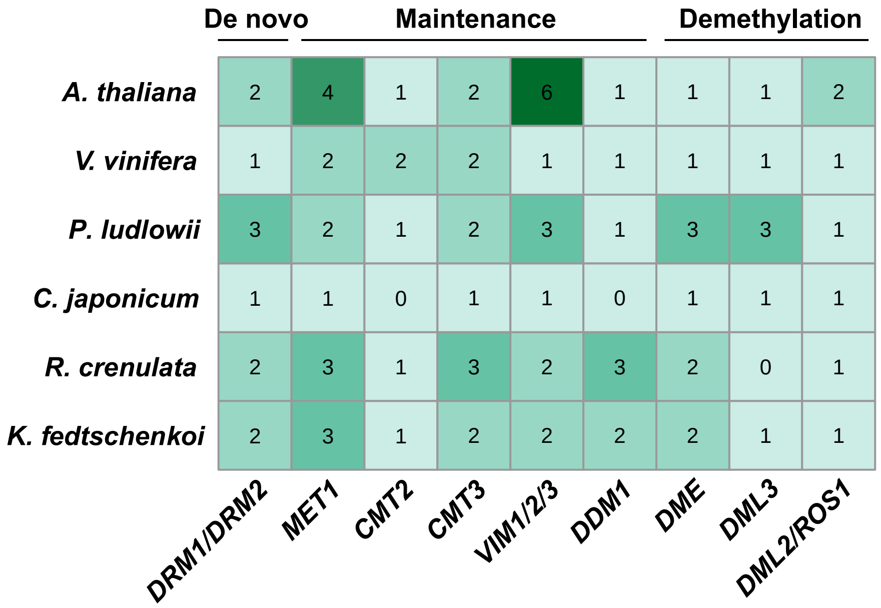

Figure S17. Counts of genes involved in DNA methylation and demethylation pathways in Saxifragales and Vitales genomes. *DRM1/2: Domains Rearranged Methyltransferase 1/2*, *MET1: Methyltransferase 1*, *CMT2/3: Chromomethylase 2/3*, *VIM1/2/3: Variant In Methylation 1/2/3, DDM1: Decreased In DNA Methylation 1*, *DME: Demeter*, *DML2/3: Demeter-Like 2/3*, *ROS1: Repressor Of Silencing 1*.

	FAD4	SAD	ADS/FAD5	DES-1-LIKE	SLD	FAD6	FAD3/7/8	FAD2	Total
V. vinifera	2	9	1	1	3	1	4	2	23
K. fedtschenkoi	2	6	14	1	3	2	2	4	34
R. crenulata	1	3	7	1	4	2	2	3	23
P. ludlowii	1	10	1	1	2	1	6	7	29
P. ostii	1	10	0	2	4	1	9	5	32
C. japonicum	1	7	1	2	2	1	1	2	17
A. thaliana	3	7	9	1	2	1	3	1	27

Figure S18. Gene number of the *FAD* gene family identified in *P. ludlowii* and other six plant genomes. *FAD4*: *Fatty desaturase 4*, *SAD*: stearoyl-ACP desaturase, *ADS: ACYL-LIPID DESATURASE*, *FAD5: Fatty desaturase 5*, *SLD: SPHINGOID LCB DESATURASE 2*, *FAD6: Fatty desaturase 6*, *FAD3/7/8: Fatty desaturase 3/7/8*, *FAD2: Fatty desaturase 2*.

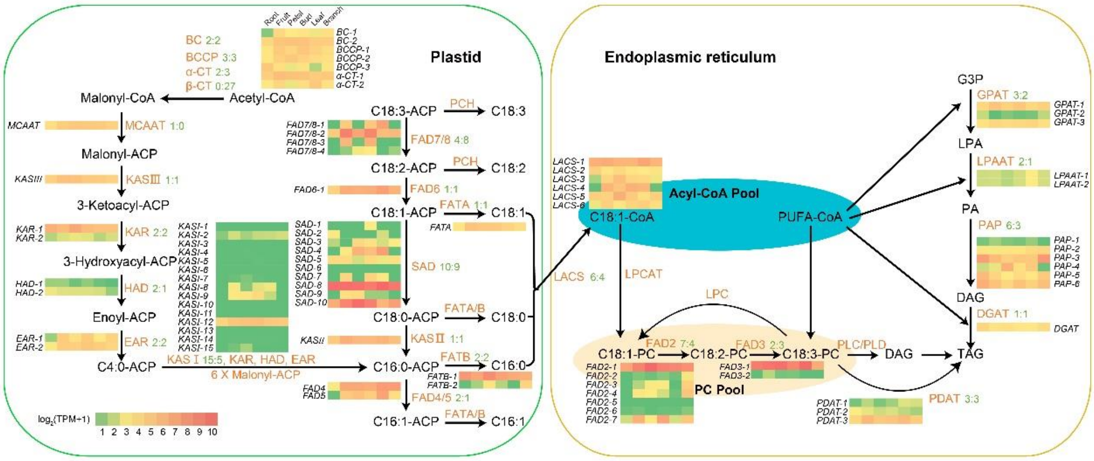

Figure S19. Expression, TE insertion, and DNA methylation of genes potentially involving the biosynthesis of fatty acids. The numbers in green indicate the copy number of corresponding genes in *P. ludlowii* and *P. ostii*. Expression level is quantified based on the TPM (Transcripts Per Million) and visualized as heatmap.

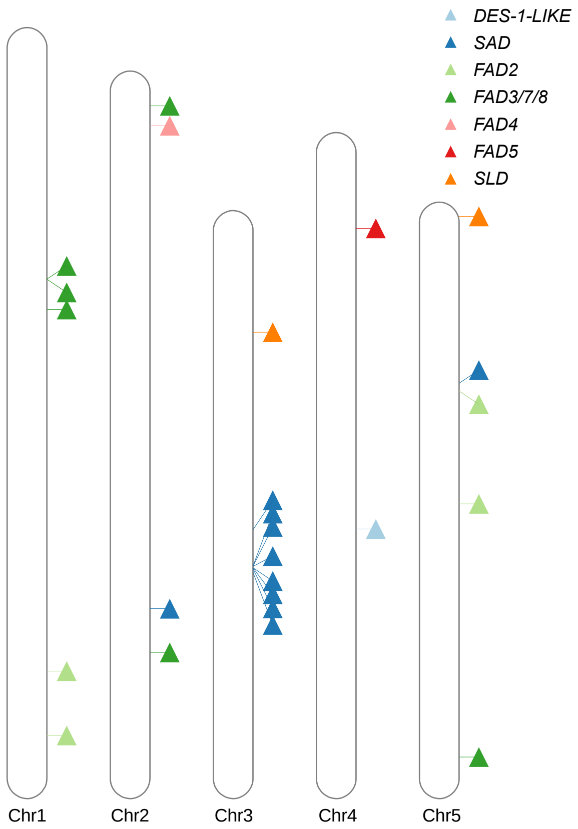

Figure S20. Distribution of FAD genes on *P. ludlowii* chromosomes.

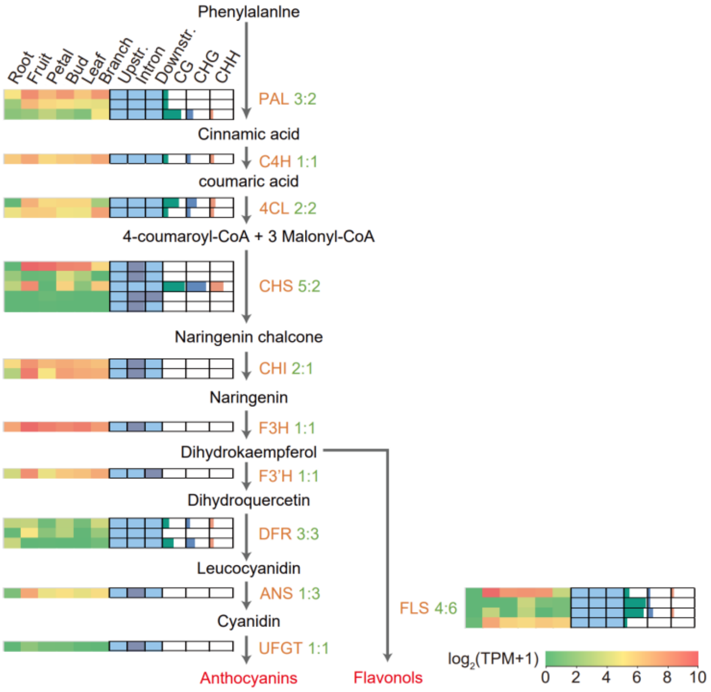

Figure S21. Expression, TE insertion, and DNA methylation of genes potentially involving flower color pathway. The numbers in green indicate the copy number of corresponding genes in *P. ludlowii* and *P. ostii*. Expression level is quantified based on the TPM (Transcripts Per Million) and visualized as heatmap. The light blue and grey rectangles indicate genes with (blue) or without (grey) TE insertion at upstream, downstream and introns. DNA methylation levels in CG, CHG, and CHH sequence context are shown by horizontal histograms.

	TPS-a1	TPS-a2	TPS-b	TPS-c	TPS-d	TPS-e/f	TPS-g	TPS-h	Total
S. moellendorffii	0	0	0	6	0	3	0	12	21
G. biloba	0	0	0	1	30	1	0	0	32
O. sativa	0	0	0	2	0	9	2	0	41
V. vinifera	0	24	6	2	0	3	13	0	48
K. fedtschenkoi	0	10	12	2	0	1	4	0	29
R. crenulata	0	0	4	0	0	2	0	0	6
P. ludlowii	0	30	31	3	0	4	7	0	75
P. ostii	0	34	17	1	0	2	11	0	65
C. japonicum	0	3	5	0	0	5	2	0	15
A. thaliana	28	23	6	1	0	2	1	0	33

Figure S22. Gene number of the TPS gene family identified in *P. ludlowii* and other nine plant genomes.

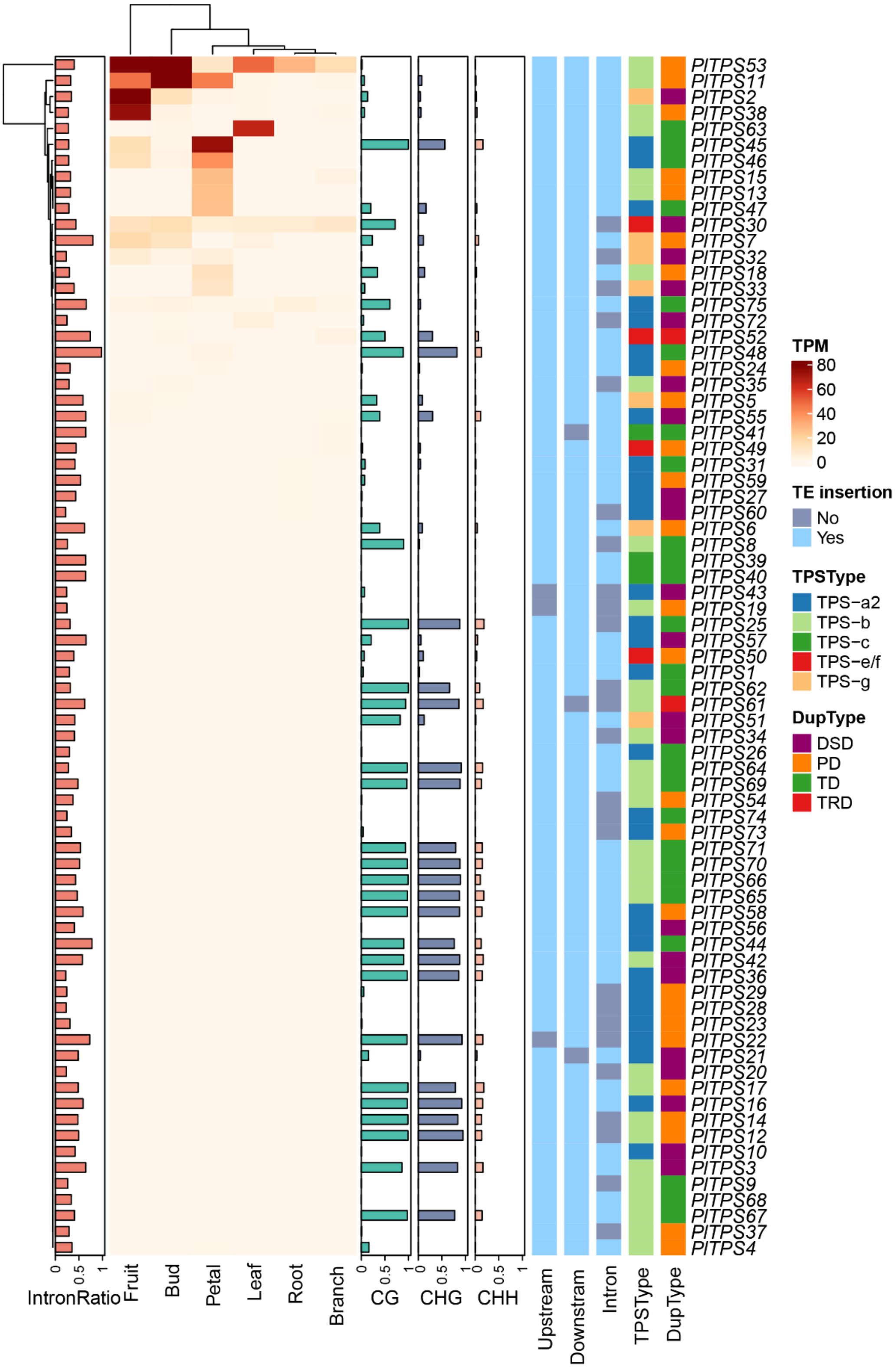

Figure S23. Expression, DNA methylation, TE insertion, TPS type, duplication type, and intron-to-gene length ratio of TPS gene family. Expression level is quantified based on the TPM (Transcripts Per Million) and visualized as heatmap with hierarchal clustering. The light blue and grey rectangles indicate genes with (blue) or without (grey) TE insertion at upstream, downstream and introns. TPS types are shown in different colors. The duplication types of the TPS gene, including DSD, PD, TD, and TRD, are also shown in different colors. Intron Ratio refers to the ratio of intron length to gene length.

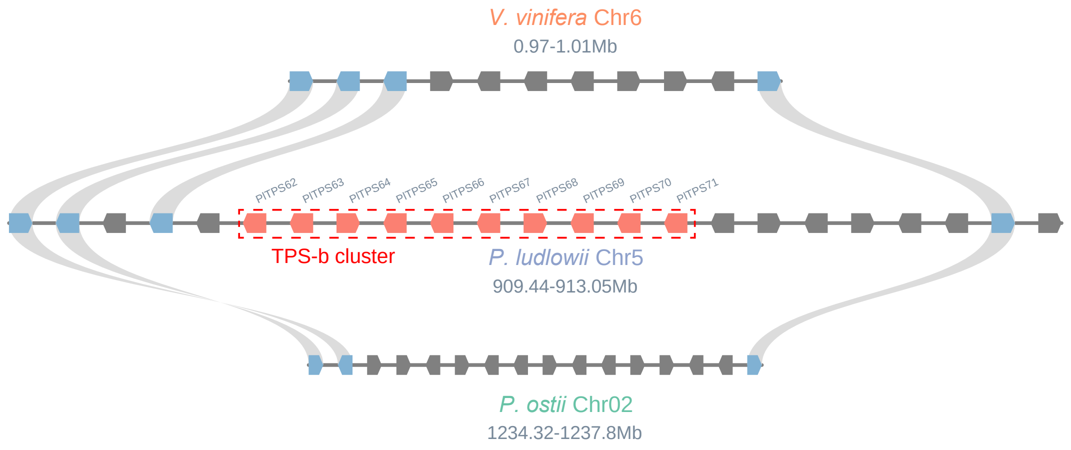

Figure S24. Micro-synteny of one tandem TPS-b gene cluster among *P. ludlowii*, *P. ostii*, and grape (*V. vinifera*) genomes. Orthologous genes (*TPS* excluded) are indicated with blue triangle and linked. The direction of the triangle represents the strand of the genes. TPS gene cluster is indicated by dotted box.

Supplementary References

1. Wan T, Liu Z, Leitch IJ et al. The Welwitschia genome reveals a unique biology underpinning extreme longevity in deserts. *Nat Commun.* 2021;12:4247.
